# Airway surveillance and lung viral control by memory T cells induced by COVID-19 mRNA vaccine

**DOI:** 10.1172/jci.insight.172510

**Published:** 2023-11-22

**Authors:** Brock Kingstad-Bakke, Thomas Cleven, Hailey Bussan, Boyd L. Yount, Ryuta Uraki, Kiyoko Iwatsuki-Horimoto, Michiko Koga, Shinya Yamamoto, Hiroshi Yotsuyanagi, Hongtae Park, Jay S. Mishra, Sathish Kumar, Ralph S. Baric, Peter J. Halfmann, Yoshihiro Kawaoka, M. Suresh

**Affiliations:** 1 Department of Pathobiological Sciences, University of Wisconsin-Madison, Madison, Wisconsin, USA.; 2 Department of Microbiology and Immunology, University of North Carolina-Chapel Hill, Chapel Hill, North Carolina, USA.; 3 Division of Virology, Institute of Medical Science, University of Tokyo, Tokyo, Japan.; 4 The Research Center for Global Viral Diseases, National Center for Global Health and Medicine Research Institute, Tokyo, Japan.; 5 Department of Infectious Diseases and Applied Immunology, IMSUT Hospital of The Institute of Medical Science, and; 6 Division of Infectious Diseases, Advanced Clinical Research Center, Institute of Medical Science, University of Tokyo, Tokyo, Japan.; 7 Department of Comparative Biosciences, University of Wisconsin-Madison, Madison, Wisconsin, USA.; 8 The University of Tokyo, Pandemic Preparedness, Infection and Advanced Research Center (UTOPIA), Tokyo, Japan.

**Keywords:** COVID-19, Immunology, Cellular immune response, Memory, T cells

## Abstract

Although SARS-CoV-2 evolution seeds a continuous stream of antibody-evasive viral variants, COVID-19 mRNA vaccines provide robust protection against severe disease and hospitalization. Here, we asked whether mRNA vaccine–induced memory T cells limit lung SARS-CoV-2 replication and severe disease. We show that mice and humans receiving booster BioNTech mRNA vaccine developed potent CD8 T cell responses and showed similar kinetics of expansion and contraction of granzyme B/perforin-expressing effector CD8 T cells. Both monovalent and bivalent mRNA vaccines elicited strong expansion of a heterogeneous pool of terminal effectors and memory precursor effector CD8 T cells in spleen, inguinal and mediastinal lymph nodes, pulmonary vasculature, and most surprisingly in the airways, suggestive of systemic and regional surveillance. Furthermore, we document that: (a) CD8 T cell memory persists in multiple tissues for > 200 days; (b) following challenge with pathogenic SARS-CoV-2, circulating memory CD8 T cells rapidly extravasate to the lungs and promote expeditious viral clearance, by mechanisms that require CD4 T cell help; and (c) adoptively transferred splenic memory CD8 T cells traffic to the airways and promote lung SARS-CoV-2 clearance. These findings provide insights into the critical role of memory T cells in preventing severe lung disease following breakthrough infections with antibody-evasive SARS-CoV-2 variants.

## Graphical abstract

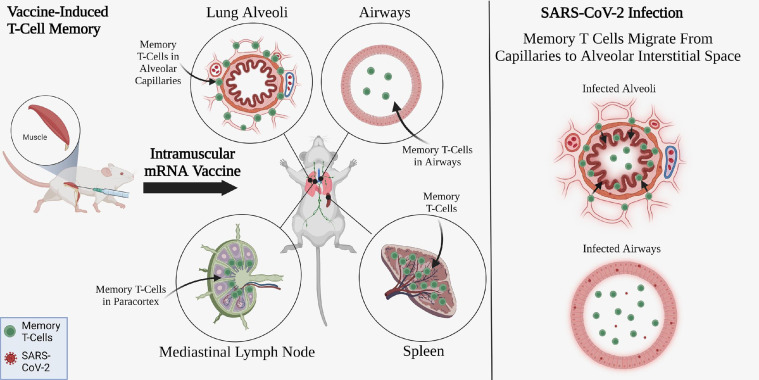

## Introduction

The rapid development of efficacious mRNA vaccines to combat the SARS-CoV-2 pandemic is one of the greatest medical and scientific achievements of the 21st century. While nucleic acid–based vaccines have been widely known and tested since the mid-1990s, no commercially available DNA or RNA vaccines existed for human use before the advent of lipid nanoparticle Moderna and Pfizer-BioNTech mRNA vaccines expressing the SARS-CoV-2 spike gene. To date, billions of doses of the COVID-19 mRNA vaccines have been given globally, and numerous studies have demonstrated their effectiveness in reducing severe disease outcomes and death (1–4).

Unlike many inactivated and subunit-based vaccines, mRNA vaccination expresses antigen within cells, leading to robust T cell responses, particularly for CD8 T cells, which serve to recognize antigen presented from an intracellular origin. The ability to elicit a potent T cell response may be a major advantage for combating the continuous emergence of global variants, because epitopes recognized by CD4 and CD8 T cells and not antibodies are strongly conserved across variants (5). While many studies have documented that CD4 and CD8 T cells elicited by mRNA vaccines strongly correlated with more positive outcomes following infection with SARS-CoV-2, and that these T cell responses are long lived (6–13), incisive studies to determine the protective mechanisms, tissue distribution of memory T cells, or the kinetics of the T cell response at an organ-by-organ level are not possible in humans. Therefore, we developed a nontransgenic mouse model to study the defining characteristics of CD8 T cell responses induced by mRNA vaccinations and the protective mechanisms underlying the protection against SARS-CoV-2 in the lungs. We show that the CD8 T cell responses of humans and mice to the booster COVID-19 mRNA vaccine are largely similar in the peripheral blood and provide unequivocal evidence of respiratory (airways, lung vasculature, and mediastinal lymph nodes [mLN]) and systemic immunosurveillance by memory CD8 T cells that effectively reduce SARS-CoV-2 burden in lungs by CD4 T cell dependent mechanisms. These findings have provided insights into the character and anatomy of the T cell response to COVID-19 mRNA vaccine and the CD8 T cell–dependent protective mechanisms against SARS-CoV-2 control in lungs. New insights from this study have significant implications in understanding how COVID-19 mRNA vaccines reduce disease severity and hospitalizations following breakthrough infections and underscore the prominent role of memory T cells in protection against rapidly evolving antibody-evasive viral variants.

## Results

### Parenteral COVID-19 mRNA vaccination elicits potent systemic and pulmonary effector CD8 T cell responses in mice.

**Figure 1:**
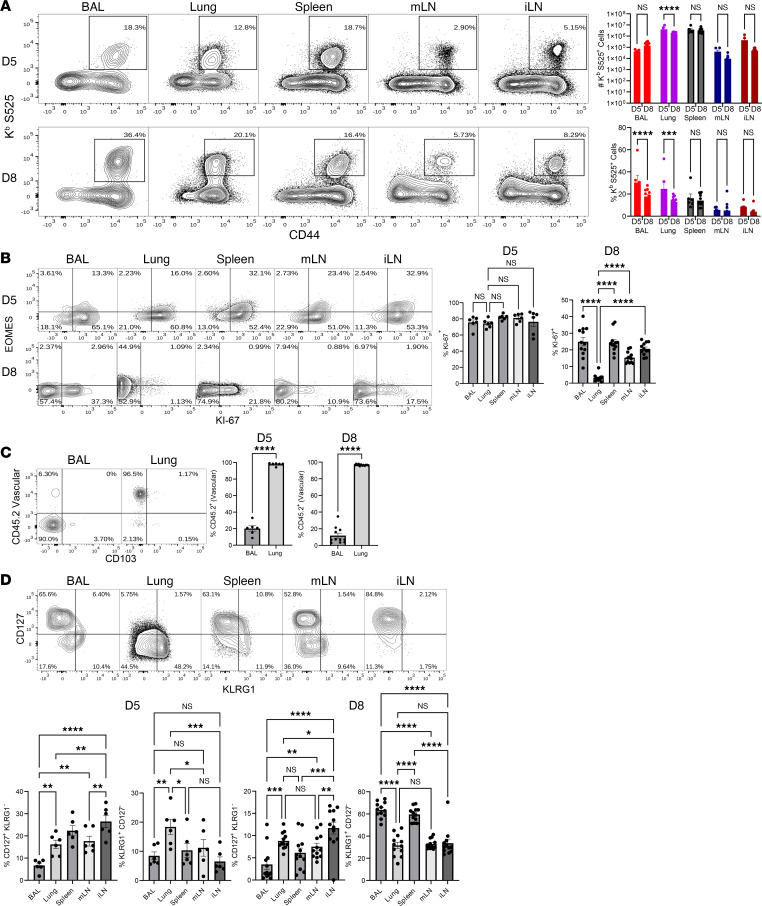
Peak CD8 T cell responses elicited by mRNA vaccination. Mice (*n* = 6) were administered twice with the BioNTech mRNA vaccine and euthanized at day 5 (D5) or D8 after the booster vaccination. Single-cell suspensions of BAL, lungs, spleen, mediastinal or inguinal lymph nodes were stained with viability dye, followed by K^b^/S525 (VNFNFNGL) tetramers in combination with antibodies to CD4, CD8, CD44, CD127, KLRG1. (**A**) Frequencies among CD8 T cells and numbers of S525-specific CD8 T cells in the indicated tissue are shown in FACS plots and graphs at D5 and D8 after booster vaccination. (**B** and **D**) FACS plots and graphs show percentages of indicated subsets among S525^+^ CD8^+^ T cells in various tissues. (**C**) To identify circulating/vascular cells in the lungs, mice were injected i.v. with fluorescent-labeled anti-CD45.2 antibodies, 3 minutes prior to euthanasia (CD45.2^+^, vascular; CD45.2^–^, nonvascular). **C** shows percentages of vascular (CD45.2^+^) and nonvascular (CD45.2^–^) cells among S525-specific CD8 T cells. Data represent 4 independent experiments. Planned comparisons were made using unpaired t test for 2-way comparisons (**A** and **C**) or Fisher’s LSD test (**B** and **D**). *, **, ***, and **** indicate significance at *P* < 0.05, < 0.005, < 0.0005, and < 0.00005, respectively. Data in each graph indicate mean ± SEM.

Previous preclinical studies with COVID-19 mRNA vaccines in mice have used doses ranging from 5 μg to 20 μg (14–17). Our pilot immunogenicity studies with the BioNTech COVID-19 mRNA vaccine in mice showed that doses of 2.5, 5.0, and 10 μg mRNA elicited comparable frequencies of antigen-specific CD8 T cells in the spleen, but responses to the 10 μg dose showed low variability (Supplemental Figure 1; supplemental material available online with this article; https://doi.org/10.1172/jci.insight.172510DS1). Hence, we chose to use a 10 μg dose for all our experiments described in this manuscript. To determine the magnitude and tissue distribution of the antigen-specific effector CD8 T cell responses elicited by the human BioNTech COVID-19 mRNA vaccine, we vaccinated cohorts of C57BL/6J (B6) mice twice (3 weeks apart) by the intramuscular (i.m.) route. At days 5 and 8 after the second dose of the vaccine, we analyzed the immunodominant K^b^-restricted CD8 T cell response to S525-532 (S525) epitope (18) in the respiratory tract (airways [broncoalveolar lavage; BAL], lungs and lung-draining mLN), spleen, and the vaccine-draining lymph node (inguinal lymph node [iLN]). At day 5 and day 8, mRNA vaccination elicited remarkably high frequencies of spike-specific CD8 T cells in all tissues measured (Figure 1A). Frequencies and numbers of S525-specific CD8 T cells at day 5 were slightly higher or similar to those at day 8, and the percentages of proliferating Ki67^+^ S525-specific CD8 T cells dropped substantially between days 5 and 8 (Figure 1B). Based on these data, we inferred that the peak of the response likely occurred in most tissues at day 5 after booster vaccination. Most notably, i.m. immunization elicited unexpectedly high frequencies and numbers of CD8 T cells in respiratory tract–associated tissues (airways, lungs, and mLN); frequencies of S525-specific CD8 T cells at days 5 and 8 ranged from 12% to 60% in BAL, 13% to 52% in lung, and 3% to 8% in mLN (Figure 1A). By performing vascular staining, we assessed whether vaccine-elicited CD8 T cells resided in the lung vasculature or parenchyma. We found that S525-specific CD8 T cells in BAL were completely excluded from vascular staining, and the majority of S525-specific CD8 T cells in the lungs were confined to the pulmonary vasculature (Figure 1C). These findings suggest that mRNA vaccines elicited a strong systemic CD8 T cell response in the spleen, draining LN, and nondraining lymph nodes. Additionally, these data suggest that mRNA vaccine elicited high levels of effector CD8 T cells in airways, pulmonary vasculature, and mLN, suggestive of regional respiratory surveillance.

Next, we examined the heterogeneity of effector CD8 T cells in various tissues, in terms of their differentiation status based on the expression of the IL-7 receptor (CD127) and the senescence marker KLRG1 — i.e., memory precursor effector cells (MPECs; CD127^hi^KLRG1^lo^) and short-lived effector cells (SLECs; CD127^lo^KLRG1^hi^) (Figure 1D). At day 5, the percentages of MPECs in lymphoid tissues spleen and LNs were in the range of 15%–25%, and these percentages dropped to 5%–15% by day 8. The drop in percentages of MPECs in lymphoid tissues was linked to a proportional increase in the percentages of SLECs between days 5 and 8 after vaccination; SLEC accumulation was more pronounced (5-fold increase) in the spleen and BAL between days 5 and 8, and this is suggestive of preferential trafficking into and/or accumulation of SLECs in lung airways. It is noteworthy that, at day 8 after vaccination, there was a stepwise increase in the MPEC frequencies between spleen, mLN, and iLN and a reciprocal and proportional reduction in the frequencies of SLECs in respective tissues. Thus, the vaccine-draining LN (iLN) appeared to support MPEC accumulation more strongly, than spleen and the nondraining lymph node (mLN).

**Figure 2:**
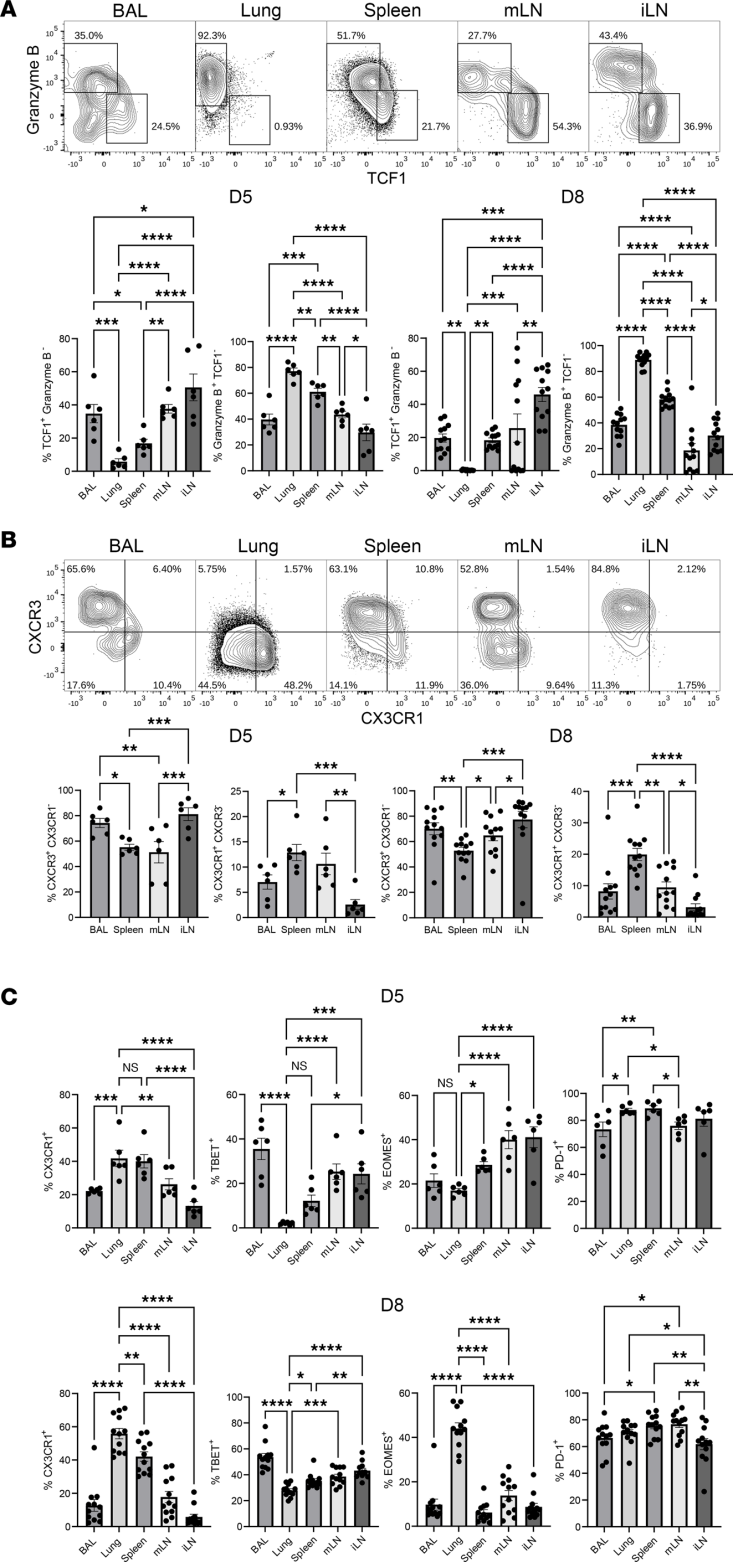
mRNA vaccine–elicited effector CD8 T cells are marked by high CXCR3 and granzyme B expression. C57BL/6 mice (*n* = 6) were vaccinated twice with monovalent BioNTech mRNA vaccine and euthanized; cells isolated from various tissues were stained as described in Figure 1, with additional antibodies to granzyme B, TCF-1, CXCR3, CX3CR1, TBET, EOMES, and PD-1. (**A**–**C**) FACS plots and graphs show percentages of indicated subsets among S525^+^ CD8^+^ T cells in various tissues at days 5 and 8 after boost. Planned comparisons were Fisher’s LSD test. *, **, ***, and **** indicate significance at *P* < 0.05, < 0.005, < 0.0005, and < 0.00005, respectively. Data in each graph indicate mean ± SEM.

To gain further insight into the differentiation of effectors and MPEC following mRNA vaccination, we analyzed the coexpression of the effector molecule granzyme B and the prosurvival transcription factor TCF-1, which is intimately linked to the development of memory T cells (19). First, it should be noted that the COVID-19 mRNA vaccine stimulated, in all tissues, robust numbers of bonafide effector CD8 T cells that expressed high levels of granzyme B (Figure 2A). Second, our analysis resolved 2 distinct subsets of effector T cells that expressed markedly different levels of granzyme B and TCF-1: granzyme B^hi^TCF-1^lo^ (effector cells; ECs) and granzyme B^lo^TCF-1^hi^ (memory precursor effectors [MPs]) (Figure 2A). Both at day 5 and 8 after vaccination, there was a graded increase in the percentages of MPs in lungs, spleen, mLN, and iLN (Figure 2A); reciprocally there was a graded decrease in the percentages of ECs in lungs, the spleen, mLN, and iLN. We validated flow cytometric quantification of TCF-1 in CD8 T cells by Western blot, which showed the expected downregulation of TCF-1 in effector CD8 T cells from the spleen of vaccinated mice, as compared with CD8 T cells in the spleen of unvaccinated mice (Supplemental Figure 2). The percentages of ECs among S525-specific CD8 T cells were higher in lung vasculature and spleen, and the percentages of MPs were highest, especially in the iLN. Interestingly, like the spleen, BAL CD8 T cells contained a mixture of ECs and MPs, but there was a conspicuous enrichment for ECs with very few MPs in the lung vasculature. These data suggest that lung vasculature might preferentially harbor ECs, and accumulation of S525-specific CD8 T cells — especially the MPs in the airways — might occur by mechanisms independent of cell trafficking from lung vasculature. As another metric for the differentiation status of effector CD8 T cells, we quantified the expression of the chemokine receptors CXCR3 and CX3CR1 (Figure 2B); elevated CX3CR1 expression has been associated with terminal differentiation of CD8 T cells (20, 21). Strikingly, 50%–80% of S525-specific CD8 T cells in all tissues expressed CXCR3. CXCR3 expression in lungs could not be assessed rigorously because enzymatic digestion of the lung tissue led to selective loss of cell-surface CXCR3 on the isolated CD8 T cells. However, lungs contained higher percentages of CX3CR1^+^ CD8 T cells than in other tissues (Figure 2C). The percentages of CXCR3^+^CX3CR1^–^ cells in iLN were higher than in spleen and mLN (Figure 2B). Taken together, S525-specific effector CD8 T cells in the lung vasculature were enriched for granzyme B^hi^TCF-1^lo^CD127^lo^ cells, and iLN contained greater percentages of granzyme B^lo^TCF-1^hi^CXCR3^hi^CD127^hi^ MP-like effector cells. Furthermore, the composition and differentiation status of effector CD8 T cells in airways mirrored splenic CD8 T cells but not those in the pulmonary vasculature. In sum, the heterogeneous population of antigen-specific CD8 T cells elicited by the mRNA vaccine consisted of bonafide effectors and memory precursors, and the relative proportions of these subsets were regulated in a tissue-specific manner. The phenotype and differentiation trajectory of effector CD8 T cells was not significantly affected by the vaccine dose, at least in the spleen (Supplemental Figure 1B).

It has become increasingly clear that the mutations accrued in the spike protein of the Omicron variant of SARS-CoV-2 have facilitated effective viral evasion of antibodies elicited by the first-generation mRNA vaccines (22–25). Therefore, an updated bivalent mRNA vaccine (containing mRNA encoding the original and the Omicron spike protein) has been used for several months as a booster vaccine. In silico analyses show that, unlike B cell epitopes, T cell epitopes in the Omicron variant spike protein might be conserved across SARS-CoV-2 variants (5). Here, we compared the immunodominant CD8 T cell responses with the original and the new bivalent vaccines. The magnitude, tissue distribution, and differentiation trajectory of effector CD8 T cells elicited by the bivalent vaccine was comparable with those induced by the original mRNA vaccine (Supplemental Figure 3, A–C). Furthermore, S525-specific effector CD8 T cells elicited by the monovalent and bivalent vaccines were functional and displayed a T Cytotoxic 1 (Tc1) polarization (Supplemental Figure 3D); upon ex vivo peptide stimulation, lung and splenic S525-specific CD8 T cells elicited by both monovalent and bivalent vaccines expressed CD40L and/or IFN-γ.

### Parenteral mRNA vaccination elicits memory CD8 T cells in the respiratory tract and lymphoid tissues.

**Figure 3:**
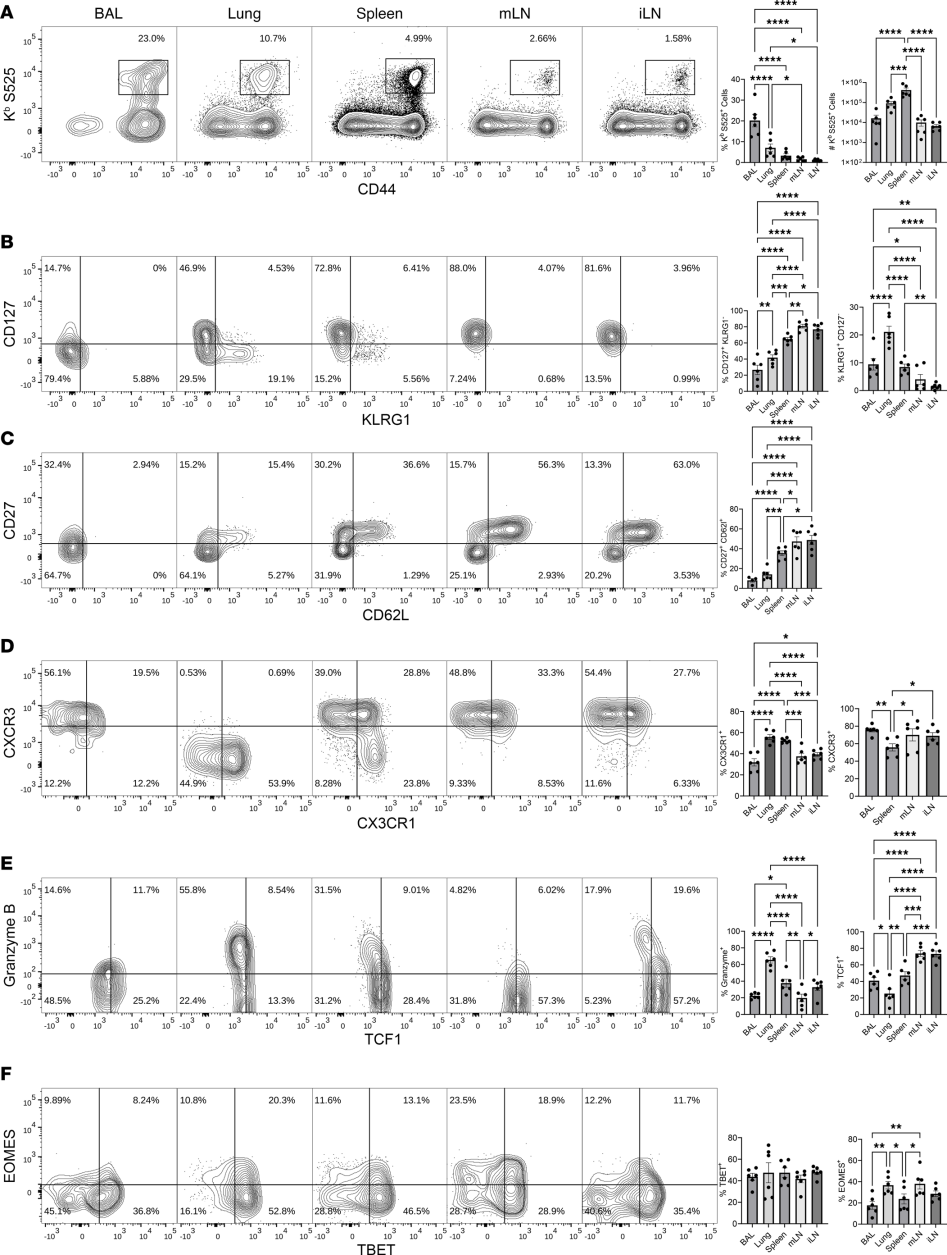
mRNA vaccine–induced mucosal and systemic CD8 T cell memory. C57BL/6 mice (*n* = 6) were vaccinated twice with monovalent BioNTech mRNA vaccine as described in Figure 1. At 96 days after booster vaccination, S525-specific memory CD8 T cells were characterized in airways (BAL), lungs, spleen, mediastinal (mLN), and inguinal (iLN) lymph nodes. Following euthanasia, organs were collected, and single-cell suspensions were stained with K^b^/S525 tetramers and antibodies for the indicated cell surface/intracellular molecules or transcription factors. (**A**) FACS plots and graphs display percentages or numbers of S525-specific CD8 T cells in various tissues. (**B**–**F**) FACS plots are gated on H-2K^b^/S525 tetramer binding cells, and the numbers are the percentages of subsets among the gated population. Data represent 2 independent experiments. Planned comparisons were made using Fisher’s LSD tests. *, **, ***, and **** indicate significance at *P* < 0.05, < 0.005, < 0.0005, and < 0.00005. Data in each graph indicate mean ± SEM.

At 96 days after booster mRNA vaccination, we found readily detectable numbers of memory CD8 T cells in the lymphoid tissues of the spleen, mLN, and iLN (Figure 3A). Surprisingly, however, we also found high frequencies of memory CD8 T cells localized to the airways and lungs, but memory CD8 T cells in the lungs were largely restricted to the pulmonary vasculature (Supplemental Figure 4, A and B). Phenotypically, lymphoid tissues contained largely CD127^hi^KLRG1^lo^ central memory CD8 T cells (Figure 3, B and C). By contrast, airway memory CD8 T cells were largely of the CD127^lo^CD27^lo^CD62L^lo^KLRG1^lo^ effector memory phenotype. Interestingly, airways and lung vasculature were enriched (>60%) for CD27^lo^CD62L^lo^ memory CD8 T cells, as compared with the spleen and lymph nodes. The chemokine receptor CXCR3 plays an important role in T cell migration to inflamed tissues expressing ligands CXCL9/10 (26) and also serves as an indicator of Tc1/Th1 effector differentiation (27). The majority of memory CD8 T cells at day 96 expressed high levels of CXCR3, and lack of CXCR3 on lung memory CD8 T cells is likely an artifact of enzymatic digestion. Like CXCR3, CX3CR1 promotes trafficking of CD8 T cells to inflamed and infected tissues via interactions with its ligand CX3CL1 expressed on endothelial cells. A greater percentage (>50%) of memory CD8 T cells in the spleen and lungs expressed elevated levels of CX3CR1, as compared with those in lymph nodes and airways, and notably, spleen contained a distinct subset of CXCR3^lo^CX3CR1^hi^ memory CD8 T cells (Figure 3D). Thus, a substantive fraction of memory CD8 T cells in circulation or in various tissues express CXCR3 and/or CX3CR1 and, hence, are poised to rapidly traffic into infected tissues such as lungs, upon infection.

Since we detected robust maintenance of memory CD8 T cells in airways and lung-draining lymph node (mLN), it was of interest to assess whether immunological milieu in these tissues led to mucosal imprinting and expression of tissue-residency markers such as CD103 and CD69. While mRNA vaccines are formulated for and only approved for parenteral use, to serve as a positive control for mucosal imprinting, cohorts of mice were vaccinated with the same mRNA vaccine by the intranasal (i.n.) route. At 96 days after i.m. vaccination, except for a very small fraction of the airway memory CD8 T cells, memory CD8 T cells in lungs, spleen, or mLN of i.m. vaccinated animals did not express CD103 and CD69 (Supplemental Figure 4C). In comparison, a substantive fraction of lung memory CD8 T cells in the i.n. vaccinated mice were found in the extravascular lung interstitium, and a fraction of memory CD8 T cells in BAL, lungs, and mLN of i.n. vaccinated mice expressed CD103 and CD69 (Supplemental Figure 4C). Thus, despite trafficking to airways and lung-draining lymph nodes, memory CD8 T cells elicited by i.m. mRNA vaccination failed to express CD103/CD69 and differentiate into classical tissue-resident memory CD8 T cells.

Notably, granzyme B–expressing effector-like memory CD8 T cells were found in all locations, but the frequencies of such effector-like memory CD8 T cells were conspicuously lower in the airways and mLN (Figure 3E). Interestingly, however, despite sharing the cell surface phenotype of effector memory cells (CD62L^lo^), memory CD8 T cells in lung vasculature but not in airways were enriched (60%) for granzyme B expression (Figure 3E). Memory CD8 T cells expressing higher levels of TCF-1 were found in greater frequencies in the lymph nodes, consistent with their CD62L^hi^CD127^hi^CD27^hi^ central memory phenotype. Expression of T-bet did not differ significantly between tissues, but EOMES expression was higher in lungs and lymph nodes (Figure 3F). In summary, the COVID-19 mRNA vaccine elicited central and granzyme B–expressing effector memory CD8 T cells, which were distributed both systemically (spleen) and locally (draining and draining lymph nodes), and in the pulmonary vasculature. Additionally, it is noteworthy that mRNA vaccine programmed a phenotypically, functionally, and transcriptionally distinct cohort of memory CD8 T cells in the lung vasculature.

Next, we determined whether the kinetics of the CD8 T cell response differed in a tissue-specific fashion. After reaching peak levels on day 5 or 8 after booster vaccination, S-specific CD8 T cells contracted in all tissues; as compared with day 5 levels, about 60%, 90%, and 76% of S-specific CD8 T cells were lost in BAL, spleen, and mLN, respectively. While there were readily detectable levels of memory CD8 T cells in airways, we did not detect any extravasation of vascular memory CD8 T cells into the pulmonary interstitium over time (Supplemental Figure 4, A and B). Effector-to-memory transition was associated with enrichment for CD62L^+^CD127^+^KLRG1^–^ memory CD8 T cells in all tissues but more prominently in spleen and lymph nodes (Supplemental Figure 4, D–F). Expression of CXCR3 and CX3CR1 did not change over time substantially (Supplemental Figure 4, G and H).

### Kinetics of mRNA-spike–specific CD8 T cells in peripheral blood of mice and humans.

**Figure 4:**
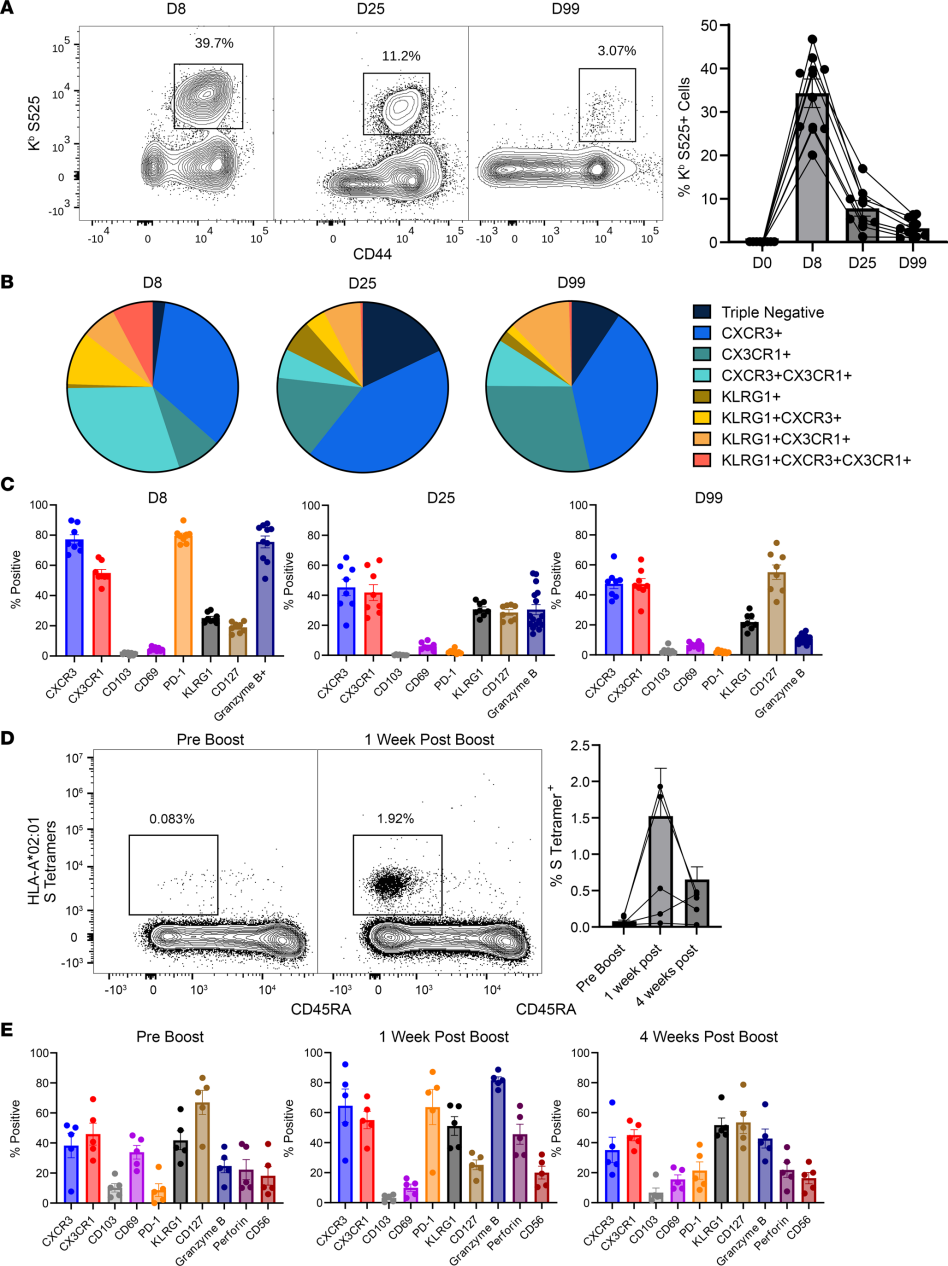
Longitudinal analysis of the kinetics and phenotypes of spike-specific CD8^+^ T cells in mouse and human PBMCs following administration of the mRNA vaccine. C57BL/6 mice (*n* = 8) were vaccinated twice with monovalent BioNTech mRNA vaccine as described in Figure 1. Human volunteers (*n* = 5) previously vaccinated with a course of the monovalent mRNA spike vaccine were given a booster of the monovalent BioNTech mRNA vaccine 180 days later. At the indicated time points before and after vaccination, peripheral blood was collected from mice or humans, and mononuclear cells were stained with K^b^/S525 tetramer (mice) or a cocktail of HLA-A*02:01 tetramers (specific to following epitopes in the S protein: 61-70, 222-230, 269-277, and 1000-1008) and antibodies to the indicated cell surface or intracellular molecules. (**A** and **D**) Graphs show longitudinal analysis of frequencies of H-2K^b^/S525-specific (mice, **A**) or S-specific (humans, **D**) tetramer binding cells among CD8^+^ T cells in PBMCs of individual mouse or humans. (**B**, **C**, and **E**) Percentages of S-specific CD8 T cells expressing the indicated molecule(s) in PBMCs of mice (**B** and **C**) or humans (**E**). Data are from 2 independent experiments. Planned comparisons were made using Fisher’s LSD tests. *, **, ***, and **** indicate significance at *P* < 0.05, < 0.005, < 0.0005, and < 0.00005, respectively. Data in each graph indicate mean ± SEM.

In most human studies, vaccine T cell responses were measured in the peripheral blood. Here, we performed longitudinal analysis of CD8 T cell responses in the circulation of mRNA-vaccinated mice and humans and asked whether: (a) murine and human CD8 T cell responses to the COVID-10 mRNA booster vaccine are similar; (b) the magnitude of CD8 T cell response in blood reflects levels in lymphoid tissues and respiratory tract; or (c) the peak CD8 T cell levels in blood forecasts the durability and magnitude of CD8 T cell memory. In mice, at the peak of the response (day 8) the frequency of S525^+^ specific CD8 T cells ranged from about 20% to 50% (Figure 4A), which was similar to frequencies detected in the spleens of vaccinated mice at this time (Figure 1A). These S525^+^ cells decreased in frequency to about 5% of total CD8 T cells by day 99. The average contraction of CD8 T cells between days 8 and 99 was 10-fold, from about 30% to 3% on average. The magnitude of the peak response correlated with the frequency of CD8 T cells at memory — i.e., mice with the highest frequency of S525-specific CD8 T cells at day 8 had the highest frequencies at day 99. Over time, the frequencies of cells expressing both CXCR3 and CX3CR1 diminished, but there was an enrichment for CXCR3^lo^CX3CR1^hi^ cells in the circulation (Figure 4B). While low levels of KLRG1^hi^ cells persisted over time, KLRG1^hi^CXCR3^hi^CX3CR1^hi^ rapidly contracted after the peak of the response, indicating that these highly differentiated cells might lose expression of one or more of these markers or that they fail to survive. Overall, S525-specific CD8^+^ T cells detected in PBMCs in mice displayed similar phenotypes to those in spleen, being CXCR3^hi^, CX3CR1^hi^, CD69^lo^, CD103^lo^, and having relatively steady levels of KLRG1, with levels of CD127 increasing over time and the peak of PD-1 expression coinciding with recent antigenic exposure (Figure 4C). However, the kinetics of Ki-67 expression in circulating mouse S525-specific CD8 T cells resembled CD8 T cells in the lung vasculature during the peak of the response (Figure 1B and Supplemental Figure 5), when nearly all cells were in a proliferative state at day 5 but proliferation dropped precipitously by day 8.

To assess the kinetics of the human CD8 T cell response to booster vaccination, we collected PBMCs from individuals prebooster and at 8 days and 4 weeks after booster vaccination. S-specific CD8 T cells were visualized by using a cocktail of peptide-loaded HLA-A-02 tetramers (to assess CD8 T cell responses to 4 epitopes restricted by the same MHC). S-specific CD8 T cells were present at low frequencies prebooster (<0.1%), but at day 8 after the booster, S-specific CD8 T cells were detected at high frequencies in 5 of 10 individuals (Figure 4D). At day 8, more than 50% of S-specific CD8 T cells expressed CXCR3, CX3CR1, KLRG1, and PD-1. Furthermore, 50%–80% of S-specific CD8 T cells expressed perforin and/or granzyme B, suggestive of effector differentiation (Figure 4E). In the ensuing 3 weeks, there was ~75% reduction in the frequencies of S-specific CD8 T cells, but the levels were significantly above the prebooster levels (Figure 4D). Like mouse PBMCs, there was a signature of high levels of CXCR3 and CX3CR1 at peak that continued into memory, low levels of CD69 and CD103, stable levels of KLRG1 with increasing levels of CD127 over time, and peak levels of PD-1 that corresponded to recent vaccination (Figure 4E). Thus, overall, the kinetics of the CD8 T cell response to a booster mRNA vaccine in humans and mice were similar (Figure 4, A–E).

### Lung-protective recall CD8 T cell responses to SARS-CoV-2 challenge.

**Figure 5:**
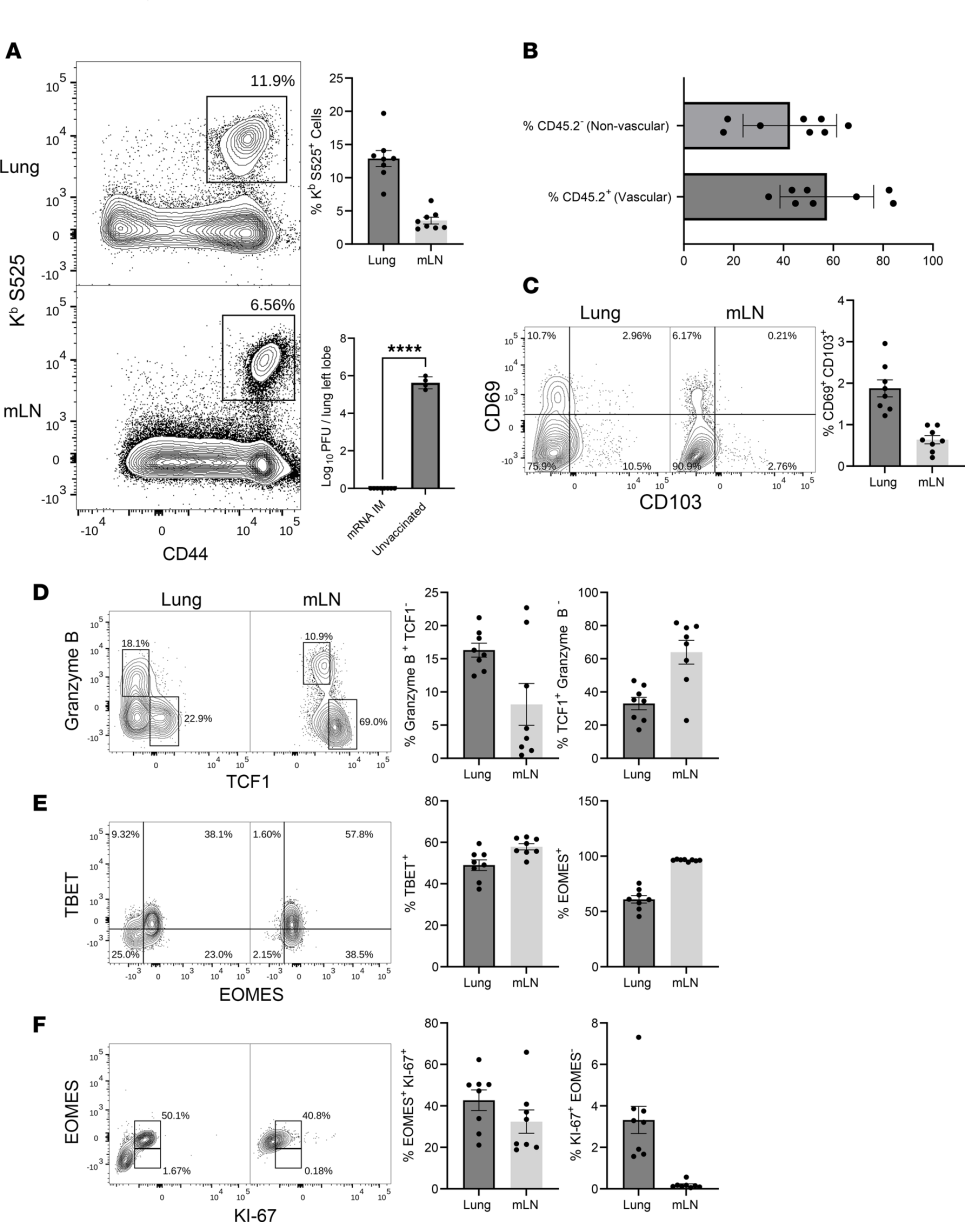
mRNA vaccine–induced T cell–dependent protective immunity to a mouse-adapted strain of SARS-CoV-2. Cohorts of 6- to 8-week-old mice (*n* = 8) were vaccinated twice with BioNTech mRNA vaccine, as described in Figure 1. At 100 days after booster vaccination, mice were challenged with the MA10/B.1.351 mouse–adapted strain of SARS-CoV-2 virus; unvaccinated mice were challenged as controls. (**A**) Viral titers and S525-specific CD8 T cells were quantified in the lungs on day 5 after challenge. (**B**) Percentages of vascular (CD45.2^+^) or nonvascular (CD45.2^–^) cells among K^b^/S525-specific CD8 T cells in lungs. (**C**–**F**) FACS plots are gated on K^b^/S525 tetramer binding CD8 T cells, and the numbers are the percentages of tetramer binding CD8 T cells within the gate or the quadrant. Two-way comparisons were made using an unpaired *t* test. ****P < 0.00005. Data in each graph indicate mean ± SEM.

Protective immunity afforded by human COVID-19 mRNA vaccines are linked to stimulation of spike-specific virus–neutralizing antibodies, which are expected to ablate infection with a homologous or a cross-reactive SARS-CoV-2 variant. However, we have shown previously that antibodies to the spike protein of the original Washington strain of SARS-CoV-2 failed to neutralize the B.1.351 variant of SARS-CoV-2 in mice (18). To limit the role for antibodies in protection and eliminate the need for K18 human ACE2 transgenic mice, we generated a mouse-adapted SARS-CoV-2 virus (MA-10/B.1.351) with the spike protein from the South African Beta variant of SARS-CoV-2 (B.1.351); MA-10 virus replicates to high levels in lungs of unmanipulated C57BL/6 mice (28). Cohorts of mice vaccinated i.m. with the monovalent COVID-19 mRNA vaccine were challenged with the MA-10/B.1.351 virus. On day 5 after viral challenge, we assessed viral load in lungs and recall CD8 T cell responses in lungs and mLN (Figure 5A). Upon viral challenge, vaccinated animals mounted strong recall CD8 T cell responses in lungs and draining lymph nodes (Figure 5A) and reduced viral burden in lungs to levels that were below the level of detection (Figure 5A). Unlike lung effector/memory CD8 T cells that were primarily intravascular and expressed little or no CD103 or CD69 prior to challenge (Supplemental Figure 4C), following viral challenge, more than 50% of S525-specific CD8 T cells displayed extravascular localization, likely to the lung interstitium, and expressed increased levels of granzyme B and CD103 and/or CD69 (13%) (Figure 5, B–D). Likewise, a subset of S525-specific CD8 T cells in the lung-draining lymph nodes expressed granzyme B (Figure 5D). Lung CD8 T cells expressed effector-driving transcription factors T-bet and/or EOMES, but all S525-specific CD8 T cells in draining lymph nodes were EOMES^+^ (Figure 5E). Interestingly, all proliferating (Ki67^+^) cells in lungs and lymph nodes expressed high levels of EOMES (Figure 5F). On average, 30% and 70% of S525-specific CD8 T cells expressed the prosurvival transcription factor TCF-1 in lungs and lymph nodes, respectively (Figure 5D). Thus, SARS-CoV-2 control in lungs of mRNA-vaccinated mice was associated with accumulation of proliferating granzyme B^+^ effector CD8 T cells in the extravascular lung tissue. Seven percent to 9% of lung CD8 T cells produced IFN-γ following ex vivo stimulation with the S525 peptide (Supplemental Figure 6), and the cytokine-producing cell frequencies mirror frequencies of MHC I tetramer^+^ S525-specific CD8 T cells (~12%); both vascular and extravascular CD8 T cells expressed IFN-γ upon stimulation.

Next, we carefully assessed the kinetics of lung viral control and pulmonary recall CD8 T cell responses at days 1, 3, and 5 after SARS-CoV-2 challenge of vaccinated mice (Supplemental Figure 7). As shown in Supplemental Figure 7A, lung viral load in vaccinated mice was ~3 log_10_ lower than in unvaccinated mice within 1–3 days after viral challenge, and by day 5, infectious virus had been almost completely eliminated in lung. Rapid viral control in lungs was associated with expeditious increase in the frequencies of granzyme B–expressing S525-specific CD8 T cells by day 1 after challenge, as compared with frequencies of memory CD8 T cells (Figure 3A). Strikingly, while almost all memory CD8 T cells are found intravascularly in the lungs prior to challenge (Supplemental Figure 7A), 40%–60% of S525-specific CD8 T cells were found localized to the extravascular tissue within 1–3 days after challenge (Supplemental Figure 7A), suggestive of rapid extravasation of vascular memory CD8 T cells into the infected lungs. Furthermore, extravascular but not intravascular S525-specific CD8 T cells in lungs of virally challenged mice displayed CD69 expression, indicative of viral antigen recognition (Supplemental Figure 7B); CD69 expression was highest at day 1 but tapered off as viral load decreased in the lungs. Consistent with the report that KLRG1^hi^ CD4 T cells have reduced ability to migrate out of the lung vasculature (29), we found higher KLRG1 expression on vascular CD8 T cells and enrichment for KLRG1^lo^ CD8 T cells among the extravascular S525-specific CD8 T cells in the lungs following viral challenge (Supplemental Figure 7B). Taken together, data in Supplemental Figure 7 suggest that rapid SARS-CoV-2 control in lungs of mRNA-vaccinated mice was associated with expeditious accumulation of KLRG1^lo^ granzyme B–expressing effector CD8 T cells in the extravascular lung tissue.

**Figure 6:**
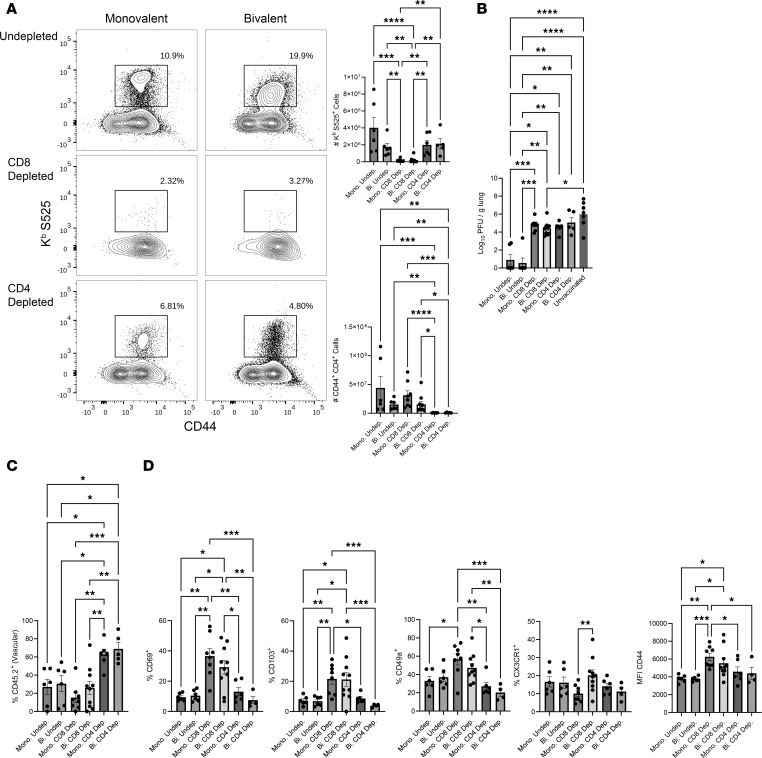
CD8 and CD4 T cells are necessary for mRNA vaccine–induced protective immunity to SARS-CoV-2 in lungs. Cohorts of 6- to 8-week-old B6 mice (*n* = 5–8) were vaccinated twice with the BioNTech monovalent (mono) or the bivalent (bi) mRNA vaccine, as described in Figure 1. At 160 days after booster vaccination, mice were treated i.n. and i.v. with anti-CD4 or anti-CD8 antibodies before and during challenge with the MA10/B.1.351 strain of SARS-CoV-2. On the day 5 after viral challenge, lung cells were stained with K^b^/S525 tetramers and antibodies to CD8, CD4, and CD44. (**A**) FACS plots are gated on total CD8 T cells. Graphs show number of S525-specific CD8^+^ and activated (CD44^+^) CD4 T cells in lungs on day 5 after challenge. (**B**) Graph shows SARS-CoV-2 titers in lungs. (**C**) Graph shows percentages of K^b^/S525-specific CD8 T cells that were found in the lung vasculature or (**D**) expressed CD69, CD103, CD49a, CD44, or CX3CR1 in lungs of virally challenged mice. Planned comparisons were made using Fisher’s LSD (**A**, **C**, and **D**) or Brown-Forsythe and Welch tests (**B**). *, **, ***, and **** indicate significance at *P* < 0.05, < 0.005, < 0.0005, and < 0.00005, respectively.

Next, to delineate the role of memory CD4 or CD8 T cells in protection against lung SARS-CoV-2 infection, we vaccinated mice with the monovalent or the bivalent vaccine, and depleted CD4 or CD8 T cells, just prior to a challenge with the MA-10/B.1.351 virus. At day 5 after viral challenge, there were strong recall CD8 T cell responses in lungs of undepleted and CD4 T cell–depleted vaccinated mice (Figure 6A), but not in CD8 T cell–depleted mice. Note the loss of S525-specific CD8 T cells and activated CD4 T cells in lungs of CD8 T cell–depleted and CD4 T cell–depleted mice, respectively. As shown in Figure 6B, viral burden in lungs of undepleted vaccinated mice was 5 logs lower than in lungs of unvaccinated controls. Remarkably, depletion of CD4 or CD8 T cells resulted in loss of viral control and high viral titers in lungs, as compared with undepleted vaccinated mice. Taken together, these findings demonstrate that memory CD4 and CD8 T cells induced by mRNA vaccination play an essential role in reducing SARS-CoV-2 viral burden in lungs.

The lack of viral control in CD4 T cell–depleted mice cannot be explained by diminished frequencies of S-specific CD8 T cells in lungs (Figure 6A). Therefore, next, we hypothesized that memory CD4 T cells promoted SARS-CoV-2 control in lungs by facilitating the extravasation of systemic memory CD8 T cells from the vasculature into the pulmonary interstitium. Intravascular staining demonstrated that > 60% of S-specific CD8 T cells in the lungs of undepleted virally challenged mice were found in the extravascular pulmonary interstitium (Figure 6C). In striking contrast, in CD4 T cell–depleted mice, up to 80% of S-specific CD8 T cells were stalled in the lung vasculature, and interestingly, CD4 depletion did not appear to substantially alter expression of either CD69, CD103, CD49a, CX3CR1, or CD44, relative to undepleted mice (Figure 6D); thus, expression of molecules that promote tissue residency or trafficking were minimally altered in CD4 T cell–depleted mice (30–33). These data demonstrate the critical importance of CD4 T cells in facilitating memory CD8 T cell extravasation into lung interstitium following viral challenge.

### Vaccine-elicited splenic memory CD8 T cells traffic to airways and control SARS-CoV-2 in lungs.

**Figure 7:**
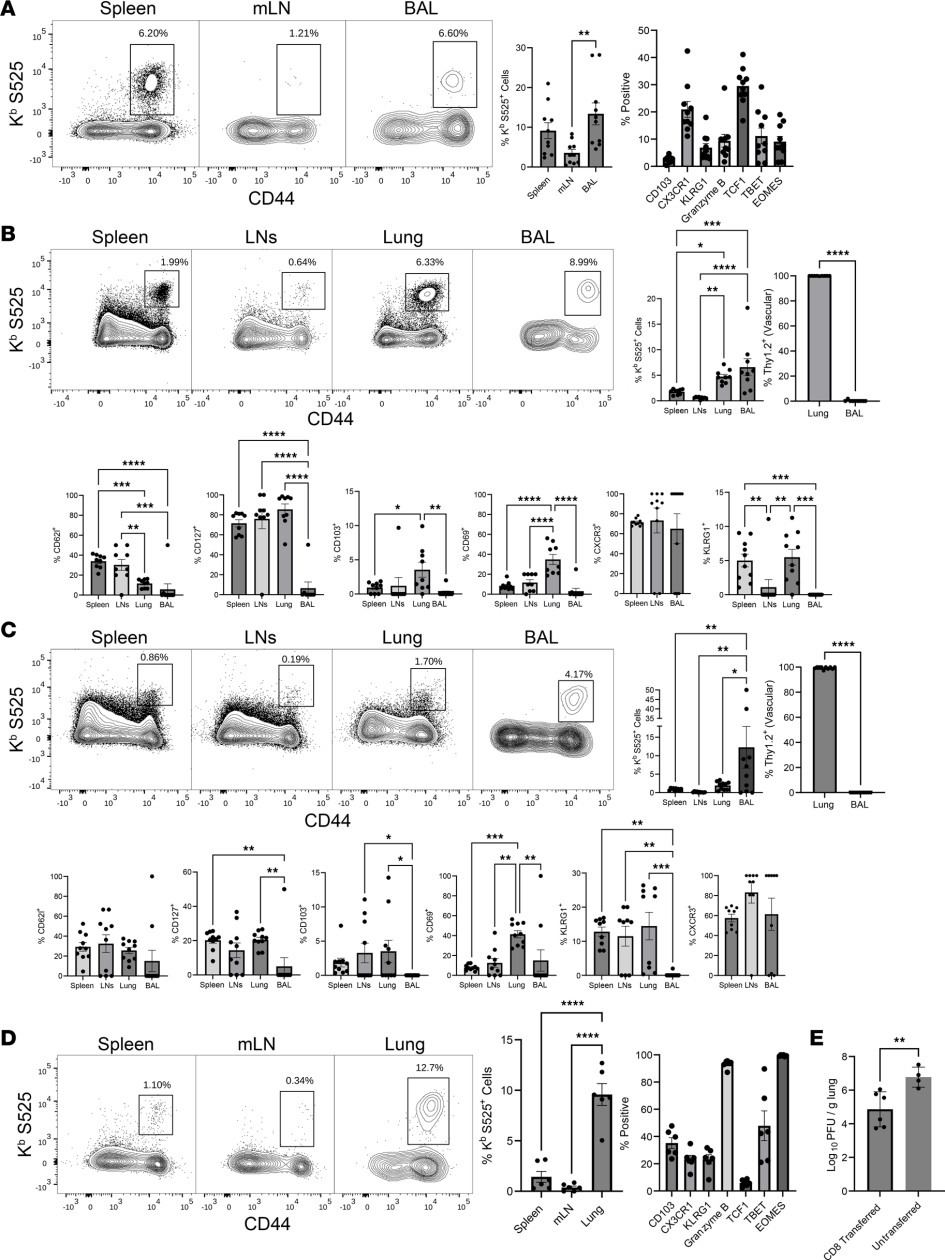
Vaccine-elicited splenic memory CD8 T cells localize to airways and lymphoid tissues and to protect against SARS-CoV-2 in lungs. Cohorts of 6- to 8-week-old CD45.2^+^ C57BL/6 mice (*n* = 5–10) were vaccinated twice with BioNTech mRNA vaccine, as described in Figure 1. (**A**) At 100 days after booster vaccination, frequencies of K^b^/S525-specific CD8 T cells were quantified in spleens, LNs, and BAL by flow cytometry; FACS plots are gated on total CD8 T cells. (**B** and **C**) CD8 T cells purified from spleens of vaccinated mice (from **A**) were adoptively transferred into congenic CD45.1 mice (*n* = 4-5). At 8 (**B**) and 30 (**C**) days after adoptive cell transfer, the frequencies and phenotype of donor CD45.2^+^ K^b^/S525-specific CD8 T cells in spleen, lymph nodes, lung, and BAL were quantified by flow cytometry. FACS plots in **B** and **C** are gated on CD45.2^+^ CD8 T cells. (**D** and **E**) At 45 days after adoptive cell transfer, mice were challenged with the MA10/B.1.351 mouse adapted strain of SARS-CoV-2 virus; unvaccinated mice were challenged as controls. (**D**) On the fifth day after viral challenge, the K^b^/S525-specific CD8 T cells in lungs were analyzed by flow cytometry. FACS plots are gated on donor CD45.2^+^ CD8 T cells. (**E**) Graph show viral titers in lungs of mice that received memory CD8 T cells (CD8 transferred) or mice that did not receive any cells (untransferred). Planned comparisons were made using Fisher’s LSD (**A–D**), or unpaired 2-tailed *t* tests for 2-way comparisons (**B** and **C** for Thy1.2+ Vascular graphs, and **E**). *, **, ***, and **** indicate significance at *P* < 0.05, < 0.005, < 0.0005, and < 0.00005, respectively. Data in each graph indicate mean ± SEM.

To reiterate, spike-specific CD8 T cells elicited by mRNA vaccination were detected in the lung vasculature but also unexpectedly to a high degree in the airways (BAL) of mice. It was of interest to determine whether memory CD8 T cells in the airways are descendants of their systemic splenic counterparts. To address this question, at 100 days after vaccination, CD8 T cells (containing 50,000–100,000 S525-specific memory CD8 T cells) purified from spleens of CD45.2 vaccinated mice were adoptively transferred into naive congenic CD45.1 C57BL/6 mice. We confirmed that spleen, BAL, and mLN of donor mice contained S525-specific CD8 T cells that were similar in frequencies and phenotype to those described in Figure 3A (Figure 7A). Cell-recipient mice were euthanized 7 and 30 days after adoptive transfer to examine the tissue distribution and phenotypes of donor memory CD8 T cells. Of great interest, at day 7 following transfer, high frequencies of donor CD45.2^+^ S525^+^ CD8 T cells were detected in airways of these mice, as measured in BAL (Figure 7B), and these cells persisted to high levels for at least 30 days (Figure 7C). Donor CD45.2^+^ S525^+^ CD8 T cells were also readily detected in lungs, mLN, and spleens, at both day 7 and day 30, and donor memory CD8 T cells localized to the lung vasculature (Figure 7, B and C). These data suggest that splenic memory CD8 T cells induced by i.m. mRNA vaccination can migrate and potentially perform immunosurveillance in the airways.

Next, we investigated whether adoptively transferred splenic memory CD8 T cells can reduce SARS-CoV-2 load in lungs. At 100 days after mRNA vaccination, CD8 T cells were purified from spleen and adoptively transferred into congenic CD45.1 mice. Thirty days after adoptive transfer, recipient mice were challenged with the MA-10/B.1.351 virus. At 5 days after challenge, we found elevated numbers of donor CD45.2^+^ granzyme B–expressing S525-specific effector CD8 T cells in lungs (Figure 7D). These donor CD8 T cells in lungs also expressed high levels of effector transcription factors EOMES and T-bet. Viral burden in lungs of adoptive transfer recipients was 10- to 100-fold lower than in untransferred mice — i.e., > 90% reduction in viral load (Figure 7E). Thus, splenic memory CD8 T cells significantly (*P* < 0.05) reduced SARS-CoV-2 levels in the lungs. Next, we assessed whether mRNA vaccine–elicited memory CD8 T cells persist long-term (day 232) and retain the ability to protect against SARS-CoV-2 in the lungs. At 232 days after vaccination, in all organs examined, the frequencies and phenotypes of S525-specific memory CD8 T cells were largely comparable with those at 96 days after vaccination, except for an enrichment of central memory (CD62L^+^) phenotype cells and a reduction in the relative proportions of CX3CR1^+^ subsets at day 232 (Supplemental Figure 4, B–H, and Supplemental Figure 8, A and B). To assess memory CD8 T cell–dependent SARS-CoV-2 control in lungs, we adoptively transferred CD8 T cells purified from spleen of mRNA vaccinated (day 232) or unvaccinated mice into C57BL/6 mice, which were challenged with MA-10/B.1.351 virus. Akin to day 100 memory CD8 T cells (Figure 7E), day 232 memory CD8 T cells from vaccinated mice also significantly (*P* < 0.01) reduced viral burden in lungs, relative to CD8 T cells from unvaccinated mice (Supplemental Figure 8C). These data suggest that mRNA vaccine–elicited splenic memory CD8 T cells provide durable protective immunity to SARS-CoV-2 in the lungs.

## Discussion

Several studies have quantified circulating levels of antibody and T cell responses to COVID-19 mRNA vaccines in humans (6–11, 34, 35), but the magnitude and the character of T cell responses in tissues including lungs and lymphoid tissues remain unknown. While a growing body of evidence suggests that mRNA vaccines can elicit potent cellular responses (6–11, 34, 35), the role of memory CD4 or CD8 T cells in mRNA vaccine–induced protection is unclear. Here, using nontransgenic immunocompetent C57BL/6 mice, we have systematically analyzed the kinetics, phenotype, and tissue distribution of COVID-19 human mRNA vaccine–elicited antigen-specific CD8 T cells as well as the role of memory CD4 and CD8 T cells in controlling SARS-CoV-2 replication in the lungs.

The kinetics of CD8 T cell responses to live viral vaccines such as the yellow fever vaccine (YFV) in humans or acute viral infections in mice (e.g., lymphocytic choriomeningitis virus [LCMV]) are composed of 3 phases: expansion, contraction, and memory (36, 37). Remarkably our studies show that CD8 T cell responses to the human COVID-19 mRNA vaccine of mice and humans mimicked the kinetics of the CD8 T cell response to acute viral infections in mice and humans. The peak of the CD8 T cell response in mice occurred at days 5–8 after booster mRNA vaccination, and 70%–90% of CD8 T cells were lost in spleen, lungs, and lymph nodes during the ensuing contraction phase. The kinetics of CD8 T cells in the circulation mirrored contraction in spleen and lymph nodes. As in acute viral infections (37, 38), longitudinal analysis of CD8 T cell frequencies showed that the frequency at the peak of the response — i.e., clonal burst size — forecasts the magnitude of CD8 T cell memory, following mRNA vaccination. Up to ~200 days after vaccination, substantive numbers of CXCR3^+^ memory CD8 T cells were detected in all tissues examined, including the pulmonary vasculature and lung airways. Furthermore, as in an acute viral infection, the CD8 T cell response to mRNA vaccine in mice is highly potent, and the frequencies of effector CD8 T cells specific to a single epitope reach 10%–20% of CD8 T cells in spleen and airways at the peak of the response. The human CD8 T cell responses to COVID-19 mRNA vaccine is robust, but the magnitude appears lower, as compared with those of SPF mice. This discrepancy could be explained by the possibility that we might be underestimating the frequencies of S-specific CD8 T cells in humans because the MHC I tetramer cocktail that we used stains CD8 T cells, recognizing epitopes restricted by only 1 MHC molecule; this likely constitutes a small fraction of all S-specific CD8 T cells elicited in the human vaccinee. It is possible that, unlike SPF mice, humans experience diverse microbial exposure throughout their lifetime, which might have dampened the CD8 T cell response to vaccines in humans (39). Therefore, to mimic the diverse microbial exposure of vaccinated humans, mRNA vaccines should be tested in “dirty” mice (specific pathogen-free mice that are exposed to pet store mice) (40) that seem to model human responses more accurately than the SPF mice. However, it is noteworthy that, despite the differences in the overall magnitude of the CD8 T cell responses, circulating S-specific CD8 T cells in SPF mice and humans displayed remarkable similarities in the kinetics, cell surface phenotype, and the subset heterogeneity among effector and memory T cells.

Notably, akin to effector CD8 T cells elicited by viral infections, CD8 T cells elicited by the mRNA vaccine differentiated into MPECs or SLECs that displayed traits of bonafide effector cells, expressed granzyme B and T-bet, and produced cytokines such as IFN-γ. This is highly significant because T cell responses triggered by acute viral infections result in durable T cell memory that lasts decades after infection, and vaccinologists have been striving to mimic such T cell programming to elicit long-term immunity (41). The current study evaluated CD8 T cell immunity in mice for only 232 days, but studies of human peripheral blood suggest that T cell memory induced by human COVID-19 mRNA vaccines is durable (7, 42–44). Mechanistically, the degree and nature of the early inflammatory response plays a crucial role in regulating the development and differentiation of effector and memory T cells following viral or intracellular bacterial infections (45, 46). Taken together, we propose that the COVID-19 mRNA vaccine likely mimics an antiviral innate inflammatory response that programs the robust differentiation of a heterogeneous pool of Type 1 effector CD8 T cells containing subsets that transition into long-lived memory T cells and display durable persistence in circulation, lymphoid tissues, and lung airways.

Typically, parenteral administration of subunit or inactivated vaccines does not elicit mucosal or tissue-resident T cell immunity (47). Surprisingly, the COVID-19 mRNA vaccine administered into the gastrocnemius muscle in the hind limbs of mice elicit high numbers of antigen-specific CD8 T cells in spleen, lungs, airways, mLN (nondraining LN), and iLN (draining LN), suggestive of a systemic response. The systemic dissemination of effector CD8 T cells might be explained by dispersal of the mRNA vaccine beyond the site of injection to other tissues such as spleen (48). It is also noteworthy that considerable numbers of effector CD8 T cells were found in lungs and airways, the target tissue for SARS-CoV-2. However, it is worth pointing out that the effector CD8 T cells in lungs did not display mucosal imprinting and were found exclusively within the pulmonary vasculature and not in the lung interstitium. Similar to our findings with mice, S-specific CD4 T cells are readily detectable in the lungs of humans receiving the mRNA vaccine, and such cells do not display markers of tissue residency (49). While these data suggest that effector CD8 T cells in the lung vasculature are unable to transendothelially migrate into the lung interstitium, it is unknown why effector CD8 T cells accumulate in the airways but not traffic to the lung interstitium. The differential migration of effector/memory T cells might be a sequel to the distinct hemodynamic properties and/or the specialized characteristics of the vasculature involved in the regional blood circulation. Blood supply to lungs occur via 2 different types of circulation: pulmonary circulation and bronchial circulation that supply blood to alveoli and airways, respectively. The pressure in the bronchial artery is 6 times that of the pressure in the pulmonary artery, which might assist CD8 T cells in transendothelial migration into airways. The differences in the diameter of blood vessels and expression of adhesion molecules on endothelial cells between the 2 types of arteries might underlie preferential migration of effector CD8 T cells to the airways but not to the lung interstitium (50, 51). Circulating effector CD8 T cells lack mucosal imprinting (expression of CD103 or CD49a) and, therefore, might readily egress the lung interstitium but fail to accumulate extravascularly. In concurrence with our findings, S-specific memory CD4 T cells have been reported in the lungs of mRNA-vaccinated humans (49). However, another study failed to detect memory CD8 T cells in BAL of humans or mice receiving the COVID-19 mRNA vaccine (11). This discrepancy may be explained by differences in the techniques used to visualize S-specific CD8 T cells. In our study, we used MHC I tetramers to detect CTLs specific to the immunodominant S525 epitope, whereas Tang et al. (11) used the less-sensitive functional readout such as cytokine production to identify S-specific CD8 T cells in the BAL. In our experience, a fraction of CD8 T cells in the BAL express PD-1 and are less able to display their functional attributes ex vivo; hence, they are a less reliable method to identify antigen-specific CD8 T cells. Although we can’t exclude the possibility that differentiation of effectors capable of migrating to the airways is seen in mice but not in humans, quantification of memory CD8 T cells in airways of vaccinated humans with MHC I tetramers will confirm whether vaccine-elicited memory CD8 T cells provide immune surveillance in the airways. By studying vaccinia virus–infected mice, Slütter and his colleagues reported continuous trafficking of systemic CXCR3^+^ memory CD8 T cells, as a mechanism of maintaining airway memory CD8 T cells (52). They also reported that memory CD8 T cells induced by vaccinia virus were superior to those induced by *Listeria monocytogenes* in trafficking to airways (52). It is possible that the superior trafficking of vaccinia virus–induced memory CD8 T cells to airways is linked to lung/airway virus replication and the associated mucosal programming of CD8 T cells. The persistence of airway memory CD8 T cells, especially those induced by i.m. mRNA vaccination, is entirely unexpected. These airway memory CD8 T cells did not express tissue residency markers such as CD103 and CD49a, and they are less likely to be mucosally programmed driven by dispersal of vaccine mRNA to the respiratory tissues and antigen expression in lungs or airways. Therefore, we favor the hypothesis that airway memory CD8 T cells in mRNA-vaccinated mice are descendants of circulating effector memory CD8 T cells. Indeed, we demonstrate that memory CD8 T cells in the spleen of mRNA-vaccinated mice traffic from the circulation to the airways, survive for at least 30 days, and show excellent protective and recall responses upon viral challenge. Our study provided fundamental insights into the trafficking of circulating memory CD8 T cells into airways, but we did not assess the exclusive role of airway memory CD8 T cells in protection against SARS-CoV-2 infection. Future work will assess whether intratracheal transfer of mRNA vaccine–elicited airway memory CD8 T cells provides protection against challenge with SARS-CoV-2.

Depletion of CD4 or CD8 T cells compromised SARS-CoV-2 control in lungs of mRNA-vaccinated mice. The mechanisms underlying the requirement for both CD4 and CD8 T cells in SARS-CoV-2 control might be multifaceted. We found that CD4 T cells promoted extravasation of memory CD8 T cells to SARS-CoV-2-infected lungs, but the underlying mechanisms are unknown and warrant further investigation. Previous work has shown that memory CD8 T cells fail to traffic into infected tissues without CD4 T cells (53); in that report, authors demonstrate that memory CD4 T cells traffic to the virally infected tissue first and produce IFN-γ that induces the production of chemokines as well as license recruitment of memory CD8 T cells from the circulation in a CXCR3-dependent fashion (53). We find no alterations in the expression of CX3CR1 and CD44 on S525-specific effector CD8 T cells in CD4 T cell–depleted group following viral challenge, but further studies are warranted to test: (a) the role of CXCR3 in regulating trafficking of CD8 T cells into the lungs and (b) whether the effector functions of CD4 T cells directly mediate viral control in lungs. Additionally, there is a need to investigate mutual interdependence of CD4 and CD8 T cells in SARS-CoV-2 control in lungs, as shown in models of tumor immunity, where APCs in the tumor environment are required to coactivate CD4 and CD8 T cells to reduce tumor burden (54, 55).

While several studies have documented durable T cell responses following mRNA vaccination (7, 42–44), recent work from the Davis group show that mRNA vaccine stimulate high numbers of CD8 CTLs, and such CTLs are superior to those induced by the SARS-CoV-2 infection (12). Another study showed that breakthrough SARS-CoV-2 infections elicit rapid recall responses of both CD4 and CD8 T cells, and the magnitude of CD8 T cell activation correlates with the rate of viral control (56). These data strongly suggest a role for CD8 T cells in viral control following breakthrough infections, but mechanistic experiments to assess the precise role of T cells in protection against SARS-CoV-2 in lungs in humans are challenging. Additionally, data from such studies are difficult to interpret, especially because of comorbidities and other confounding variables prevalent in the human population. In this context, by performing T cell–depletion experiments in vaccinated mice and by adoptive transfer of memory CD8 T cells into naive mice, our study provides unequivocal evidence that mRNA vaccine–elicited memory CD8 T cells are necessary and sufficient to effectively limit replication of a SARS-CoV-2 in lungs. We document that the rapid extravascular accumulation of memory CD8 T cells and their CD4 T cell–dependent migration into lungs was associated with expeditious SARS-CoV-2 control, but a major limitation of our study is that we did not exclude the role of binding and/or neutralizing antibodies in lung viral control. It is likely that rapid migration of vascular memory T cells into infected lungs is one of many immunological mechanisms that reduce disease severity and lower hospitalizations in human vaccinees infected with antibody evasive viral variants (57–59). It is paradigmatic that resident memory T cells in the airways and lungs provide constitutive immunity at mucosal barriers without the need for T cell migration from the systemic circulation. In our study, i.m. mRNA vaccination did not induce classical mucosally imprinted resident memory T cells in the airways or the lungs, and this finding is consistent with a human study that failed to detect resident memory T cells in the nose, following mRNA vaccination (60). However, following viral challenge, we find increased frequencies of virus-specific CD8 T cells in the lungs that expressed tissue residency markers such as CD103. These data suggest that systemic memory T cells induced by mRNA vaccine can differentiate into resident memory following breakthrough infections. Indeed, this interpretation is supported by a report that shows development of nasopharynx or nasal-resident memory T cells following breakthrough infection in humans (60, 61). Another limitation of our study is that we did not assess memory T cells in nose and upper respiratory tract or their role in controlling viral replication and/or transmission. It will be important to assess the biological significance of nasal- and airway-resident memory T cells in protection against emerging variants of SARS-CoV-2. Addressing this issue is of fundamental importance because we still do not know whether individuals that recover from breakthrough SARS-CoV-2 infections require further vaccinations or that the “hybrid immunity” is sufficient to provide broad mutation resistant immunity to future SARS-CoV-2 variants.

In summary, in this manuscript, we document that the human COVID-19 mRNA vaccine stimulated highly potent systemic CD8 T cell responses in humans and mice, and the magnitude of the response rivaled the exuberant CD8 T cell responses seen in acute viral infections. We show that vaccine-elicited memory CD8 T cells home to the pulmonary vasculature, lung draining lymph nodes, and airways and potentially perform regional immunosurveillance in the respiratory tract. We found that memory CD8 T cells may be necessary and sufficient to limit SARS-CoV-2 replication in lungs. In summary, our studies highlight the nonredundant function of memory CD8 T cells in protection against SARS-CoV-2, and ascribe a prominent role for memory T cells in limiting severe disease and hospitalization following breakthrough infections.

## Methods

### Experimental animals.

Seven- to 12-week-old male and female B6 were purchased from The Jackson Laboratory.

### Reagents.

Reagents used in these studies are listed in Supplemental Methods and in Supplemental Table 1.

### Vaccination.

The Pfizer-BioNTech monovalent (BNT162b2 [Original]) or the bivalent vaccines (or BNT162b2 [Original/Omicron BA.4/BA.5]) were provided by the University of Wisconsin Hospitals. The mRNA vaccinations were administered i.m. (100 μL) into the gastrocnemius muscle. In some experiments, mice were anesthetized with isoflurane and vaccinated i.n. (50 μL). All mice were vaccinated twice at an interval of 3 weeks.

### Tissue processing, flow cytometry, and ex vivo cytokine analysis.

BAL, lymph nodes, spleens, and lungs harvested at necropsy were processed into single-cell suspensions and stained for cellular factors, as previously described (62) (Supplemental Methods) or used in Western blot assays. For Western blot assays, primary antibodies used were anti–β-actin (Cell Signaling Technology, mouse mAb, clone 8H10D10, 1:4,000 dilution) and anti–TCF-1 (Cell Signaling Technology, rabbit mAb, clone C63D9, 1:1,000 dilution).

### Cells and viruses.

The MA-10/B.1.351 virus was derived by reverse genetics as described previously (28). Briefly, the SARS-CoV-2 MA10/B.1.351 virus was derived from an infectious clone of SARS-CoV-2 MA10 genetically engineered to replace the mouse-adapted WA spike with the native spike from the B.1.351 virus, which can bind to mouse ACE2 receptor. Viruses were cultured and tittered as described in Supplemental Methods.

### Viral challenge and adoptive cell transfer.

To induce MA-10/B.1.351 SARS-CoV-2 infection, mice were infected with 1 × 10^4^ PFU by the i.n. route. Recall responses and viral titers were assessed by euthanizing mice 5 days after infection. To assess the role of CD4 T cells and CD8 T cells in protective immunity, mice were administered 200 μg of anti-CD4 (BioXCell, GK1.5) or anti-CD8 antibodies (BioXCell, 2.43) i.v. and i.n. at days –5, –3, –1, 1, 3, and 5, relative to challenge as indicated. For adoptive-transfer studies, spleens were harvested from CD45.2-vaccinated mice at the indicated time after vaccination, processed into single-cell suspensions, then enriched for CD8 T cells using a negative selection kit (Miltenyi Biotec, 130-104-075). Four million to 5 million CD8 T cells purified from spleens of CD45.2 mRNA-vaccinated mice (purity > 90%) were transferred to congenic CD45.1 mice by retroorbital i.v. injection.

### Human clinical samples.

After informed consent was obtained, peripheral blood was collected from COVID-19 vaccinees prior to the third vaccination, 8 days after the third vaccination, and 4 weeks after the third vaccination. Detailed information of human samples is described in Table 1.

### Analysis of human samples.

Peripheral blood mononuclear cells (PBMCs) were isolated from blood obtained from COVID-19 vaccinees by using Leucosep Tubes with Porous Barrier (Greiner Bio-One). Cells were then incubated with Ghost Dye Red 780 (Cytek Biosciences) and stained with cocktail of HLA-A*02:01 tetramers (specific to following epitopes in the S protein: 61-70, 222-230, 269-277, and 1000-1008, NIH Tetramer Core Facility at Emory University) and antibodies specific to human CD4 (Biolegend, clone SK3), CD8 (Biolegend, clone RPA-T8), CD45RA (Biolegend, clone HI100), CD45RO (Cell Signaling, clone UCHL1), HLA-DR (Biolegend, clone L243), CXCR3 (BD Pharmingen, clone 1C6/CXCR3), CD69 (Biolegend, clone FN50), PD1 (Biolegend, clone EH12.2H7), CX3CR1 (Biolegend, clone 2A9-1), CD103 (Thermo Fisher, clone B-Ly7), CCR7 (BD Pharmingen, clone 3D12), CD56 (Biolegend, clone HCD56), KLRG1 (Biolegend, clone 14C2A07), and CD127 (BD Pharmingen, clone HIL-7R-M21. Antibodies against intracellular targets (Supplemental Table 1) were used for staining using the eBioscience Foxp3/Transcription Factor Staining Buffer Set (eBioscience). Data were acquired with CytoFLEX S (Beckman Coulter), and data analysis was performed using FlowJo software.

### Statistics.

Statistical analyses were performed using GraphPad software 9.0. Planned comparisons were made using a 1-way ordinary ANOVA with multiple comparisons (Fisher’s least-significant difference [LSD]) in group comparisons that did not have significantly different SDs as determined by Brown-Forsythe and Bartlett’s tests; if significantly different, multiple comparisons were made using a Brown-Forsythe and Welch test. Two way comparisons were made using an 2-tailed unpaired *t* test. *P* < 0.05 was considered significant. Data in each graph are shown as mean ± SEM.

### Study approval.

All experiments were reviewed and approved by the University of Wisconsin School of Veterinary Medicine Animal Use and Care Committee. For human studies, the research protocol was approved by the Research Ethics Review Committee of the Institute of Medical Science of the University of Tokyo (approval no. 2020-74-0226).

### Data availability.

Data are made available upon request, and all data within graphs are contained within the Supporting Data Values file.

## Author contributions

BKB, TC, HB, HP, RU, BLY, JM, and MS designed, performed, and analyzed experiments. PH provided critical reagents and assisted with experimental planning. KIH, MK, SY, and HY contributed to collection and preparation of human clinical samples. BKB and MS wrote the manuscript, which was proofread by all authors. SK, RSB, and YK provided conceptual input and mentored researchers in their laboratories. Co–first authorship and authorship order was determined by the contributions of the 2 authors to concept development, experimentation, data collection and analysis, and manuscript preparation.

## Supplementary Material

Supplemental data

Supporting data values

## Figures and Tables

**Table 1:**
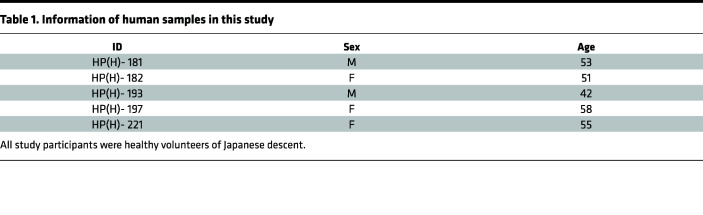
Information of human samples in this study

## Version 1 10/05/2023

In-Press Preview

## Version 2 11/22/2023

Electronic publication
