# Supplementary material for: Airway surveillance and lung viral control by memory T cells induced by COVID-19 mRNA vaccine
Source: JCI Insight. 2023 Nov 22;8(22):e172510. doi: 10.1172/jci.insight.172510 (PMC10721330; doi:10.1172/jci.insight.172510)
Supplement: Supplemental data [file jciinsight-8-172510-s069.pdf]

Supplemental Figure 1. Dose responses of S525$^+$ CD8$^+$ T cell responses to mRNA vaccination. Mice were vaccinated once with the indicated doses of the monovalent BioNTech® mRNA vaccine as in Fig. 1. At 8 days after vaccination, K^b/S525-specific CD8 T cells were characterized in spleens. (A) FACS plots and graphs show frequencies of K^b/S525-specific CD8 T cells in spleen. (B) FACS plots and graphs show percentages of the indicated cellular subsets among S525$^+$ CD8$^+$ T cells in spleens. Planned comparisons were made using Fisher's LSD (A-B). *, **, ***, and **** indicate significance at $P<0.05$, 0.005, 0.0005 and 0.00005 respectively. Data in each graph indicate mean ± SEM.

Supplemental Figure 2. Authentication of flow-cytometry protein quantification of TCF-1 by western blot. Mice (n= 5) were vaccinated twice with the monovalent BioNTech® mRNA vaccine as in Fig. 1. Eight days after vaccination, CD8 T cells (>90% pure) were isolated from spleens of vaccinated and unvaccinated mice by negative selection, pooled, and analyzed by flow cytometry and western-blot using anti-TCF-1 and anti-β-actin antibodies. (A) Blots show staining specific to TCF-1 or β-actin run in duplicate. The flow cytometry histogram shows TCF-1 levels in gated live, singlet CD8 T cells. Graph shows the mean ratio for both lanes of normalized densitometry estimated values of TCF-1 to β-actinin CD8 T cells from vaccinated or unvaccinated mice. Note the increased levels of TCF-1 protein in CD8 T cells from spleen of unvaccinated mice, as compared to those in CD8 T cells from spleen of vaccinated mice, as determined by flow cytometry and western blot.

Supplemental Figure 3. Monovalent and bivalent COVID-19 mRNA vaccination elicits similar S525$^+$ CD8 T cell responses. Mice were vaccinated twice with the monovalent or the bivalent BioNTech® mRNA vaccine as in Fig. 1. At days 5 and 8 after booster vaccination K^b/S525-specific CD8 T cells were characterized in the indicated tissues. (A) Graphs show frequencies of K^b/S525-specific CD8 T cells and percentages of vascular cells among S525-specific CD8 T cells. (B-C) Graphs show percentages of the indicated cellular subsets among S525$^+$ CD8$^+$ T cells in various tissues. (D) On D5 and D8 after booster vaccinations, cells isolated from lung and spleen were cultured with the S525 peptide, recombinant IL-2, and Brefeldin A for 5 h. FACS plots are gated on CD8 T cells and data are the percentages of IFNγ and/or CD40L-expressing cells among the gated population. Data represent four independent experiments. Two-way comparisons were made using an unpaired t test. *, **, ***, and **** indicate significance at $P<0.05$, 0.005, 0.0005 and 0.00005 respectively. Data in each graph indicate mean ± SEM.

Supplemental Figure 4. Kinetics of S525-specific CD8 T cell responses to mRNA vaccination. C57BL/6 mice (n=5-12/timepoint) were vaccinated twice with monovalent BioNTech® mRNA vaccine as described in Fig. 1. At the indicated time points after booster vaccination, S525-specific memory CD8 T cells were characterized in airways (BAL), lungs, spleen, mediastinal and inguinal lymph nodes. (A) Graphs show numbers of H-2K^{b}/S525 tetramer-binding cells in the indicated organ over time, and lines indicate the % fold change relative to D5 post boost. At each time point, mice were injected with fluorochrome-labeled anti-CD45.2 antibody prior to euthanasia to label vascular cells as in Fig. 1. (B) Percentages of vascularly labeled S525-specific CD8^{+} T cells over time in BAL and lungs. (C) Cohorts of mice were vaccinated intramuscularly or intranasally with the monovalent mRNA vaccine. Upper panel in C show percentages of CD69^{+}CD103^{+} S525-specific memory CD8 T cells in various tissues at different timepoints after IM vaccination. Lower panel in C shows CD69^{+}CD103^{+} or vascular S525-specific memory CD8 T cells in lungs and BAL, following IN vaccination. (C-H) Graphs show percentages of the indicated cellular subsets among tetramer-binding cells in the indicated tissue over time, and lines indicate the % fold change relative to D5 post boost. Data in each graph indicate mean ± SEM. Data are from two independent experiments.

Supplemental Figure 5. KI-67 levels in mRNA elicited S525-specific CD8 T cells. C57BL/6 mice (n = 4) were vaccinated twice with monovalent BioNTech® mRNA vaccine as described in Fig. 1. At 5 and 8 days after vaccination, peripheral blood mononuclear cells (PBMCs) were collected, and mononuclear cells were stained with K^b/S525 tetramer, anti-CD44, anti-CD8 and anti-KI-67 antibody. The flow cytometry histogram shows KI-67 levels in gated live, singlet tetramer-binding CD8 T cells. The graph shows frequencies of KI-67+ CD8 T cells. The Two-way comparison was made using an unpaired t test. **** indicates significance at 0.00005. Data in each graph indicate mean ± SEM.

Supplemental Figure 6. Cytokine expression from lung CD8 T cells of mRNA vaccinated mice following SARS-CoV-2 challenge Cohorts of 6-8-week-old mice (n = 5) were vaccinated and challenged as described in Fig. 1 and Fig. 4. To label vacular cells, mice were injected IV with anti-CD45.2 antibodies prior to euthanasia. On D5 after challenge, cells from lung and spleen were cultured with the S525 peptide, PMAI, or control media, and recombinant IL-2 and Brefeldin A for 5 h. FACS plots are gated on CD8 T cells and data are the percentages of IFNγ-producing cells among the gated population. Note that plots also show vascularity of CD8 T cells based on staining for CD45.2. Planned comparisons were made a Brown-Forsythe and Welch test. ** and **** indicate significance at $P < 0.005$ and 0.00005 respectively. Data in each graph indicate mean ± SEM.

Supplemental Figure 7. Early SARS-CoV-2 control in lungs of mRNA vaccinated mice. Cohorts of 6-8-week-old mice (n=5) were vaccinated twice with BioNTech® mRNA vaccine, as described in Fig 1. At **40** days after booster vaccination, mice were challenged with the MA10/B.1.351 mouse adapted strain of SARS-CoV-2 virus; unvaccinated mice were challenged as controls. (A) Viral titers and S525-specific CD8 T cells were quantified in the lungs on days 1, 3 and 5 after challenge, as well as percentages of non-vascular ($CD45.2^{-}$), Granzyme B, or $TCF\text{-}1^{+}$ cells among K^{b}/S525-specific CD8 T cells in lungs. (B) FACS plots are gated on K^{b}/S525 tetramer-binding CD8 T cells, and plots and graphs show the relative frequencies of vascular and/or non-vascular CD69+ and KLRG1+ cells among the gated cells. Planned comparisons were made using or Brown-Forsythe and Welch tests (A – viral titers), or Fisher's LSD (A-B). *, **, ***, and **** indicate significance at P<0.05, 0.005, 0.0005 and 0.00005 respectively. Data in each graph indicate mean ± SEM.

Supplemental Figure 8. Splenic long-term memory CD8 T cells protect against SARS-CoV-2 challenge. Cohorts of 6-8-week-old C57BL/6 mice (n = 5) were vaccinated twice with BioNTech® mRNA vaccine, as described in Fig 1. (A) At 232 days after booster vaccination, frequencies of K^b/S525-specific CD8 T cells were quantified in spleens, lungs, LNs and BAL by flow cytometry; FACS plots are gated on total CD8 T cells. FACS plots and graphs show percentages of indicated subsets among $S525^+$ $CD8^+$ T cells in various tissues. CD8 T cells purified from spleens of unvaccinated and vaccinated mice (from A) were adoptively transferred into naive mice (n = 5). At 7 days after adoptive cell transfer mice were challenged with the MA10/B.1.351 mouse adapted strain of SARS-CoV-2 virus; unvaccinated mice were challenged as controls. On the 5th day after viral challenge, (C) K^b/S525-specific CD8 T cells in lungs were analyzed by flow cytometry, and viral titers in lungs were quantified by plaque assay. Planned comparisons were made using Fisher's LSD or an unpaired t test (B - CD45.2 Vascular). *, **, ***, and **** indicate significance at $P<0.05$, 0.005, 0.0005 and 0.00005 respectively. Data in each graph indicate mean ± SEM.

Sup. 1

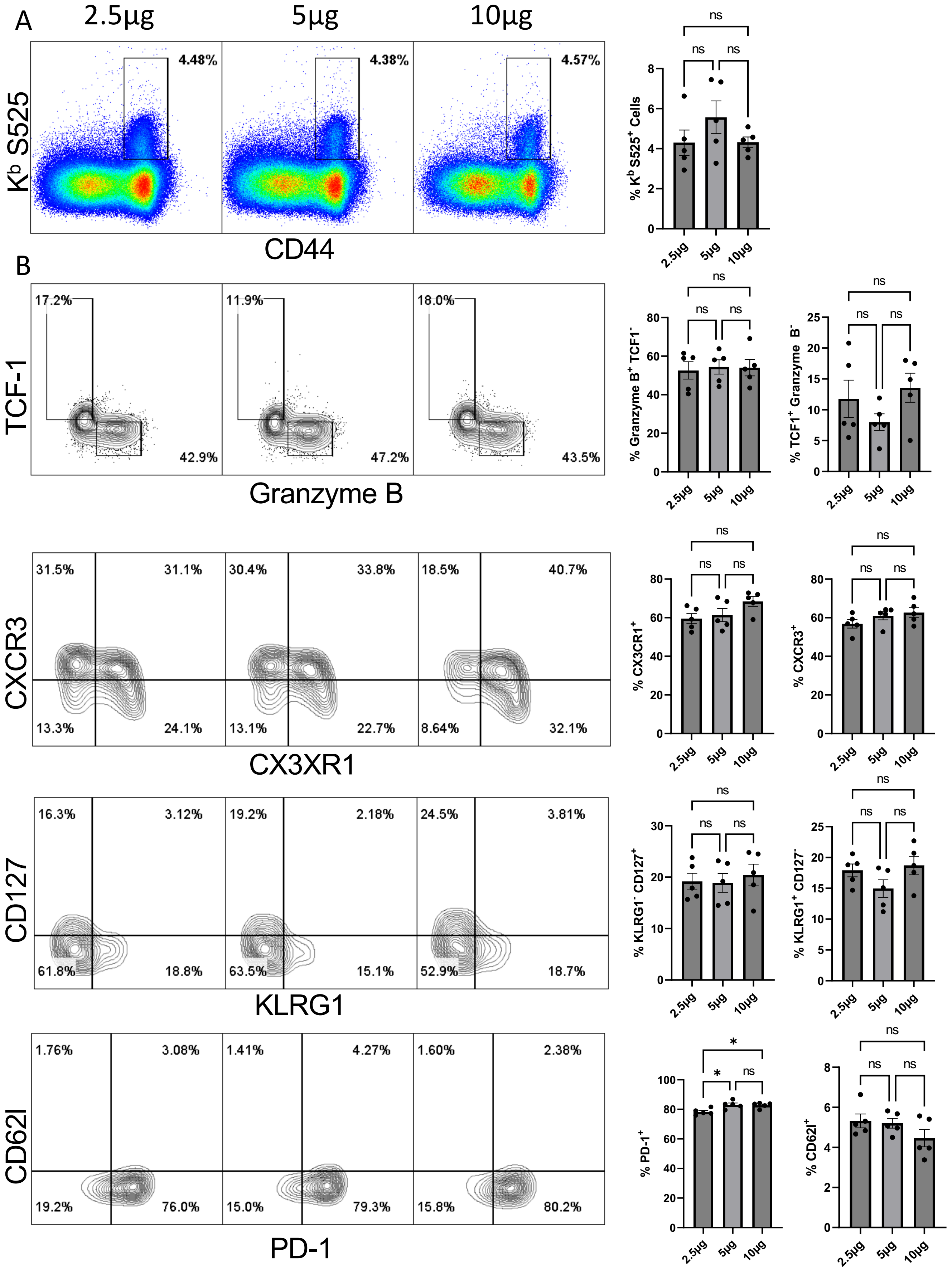

A
2.5µg
5µg
10µg
K^b S525
CD44
4.48%
4.38%
4.57%
% K^b S525+ Cells
B
TCF-1
Granzyme B
17.2%
42.9%
11.9%
47.2%
18.0%
43.5%
% Granzyme B+ TCF1-
% TCF1+ Granzyme B-
CXCR3
CX3XR1
31.5%
31.1%
13.3%
24.1%
30.4%
33.8%
13.1%
22.7%
18.5%
40.7%
8.64%
32.1%
% CX3CR1+
% CXCR3+
CD127
KLRG1
16.3%
3.12%
61.8%
18.8%
19.2%
2.18%
63.5%
15.1%
24.5%
3.81%
52.9%
18.7%
% KLRG1- CD127+
% KLRG1+ CD127-
CD62l
PD-1
1.76%
3.08%
19.2%
76.0%
1.41%
4.27%
15.0%
79.3%
1.60%
2.38%
15.8%
80.2%
% PD-1+
% CD62l+
ns
*

Sup. 2

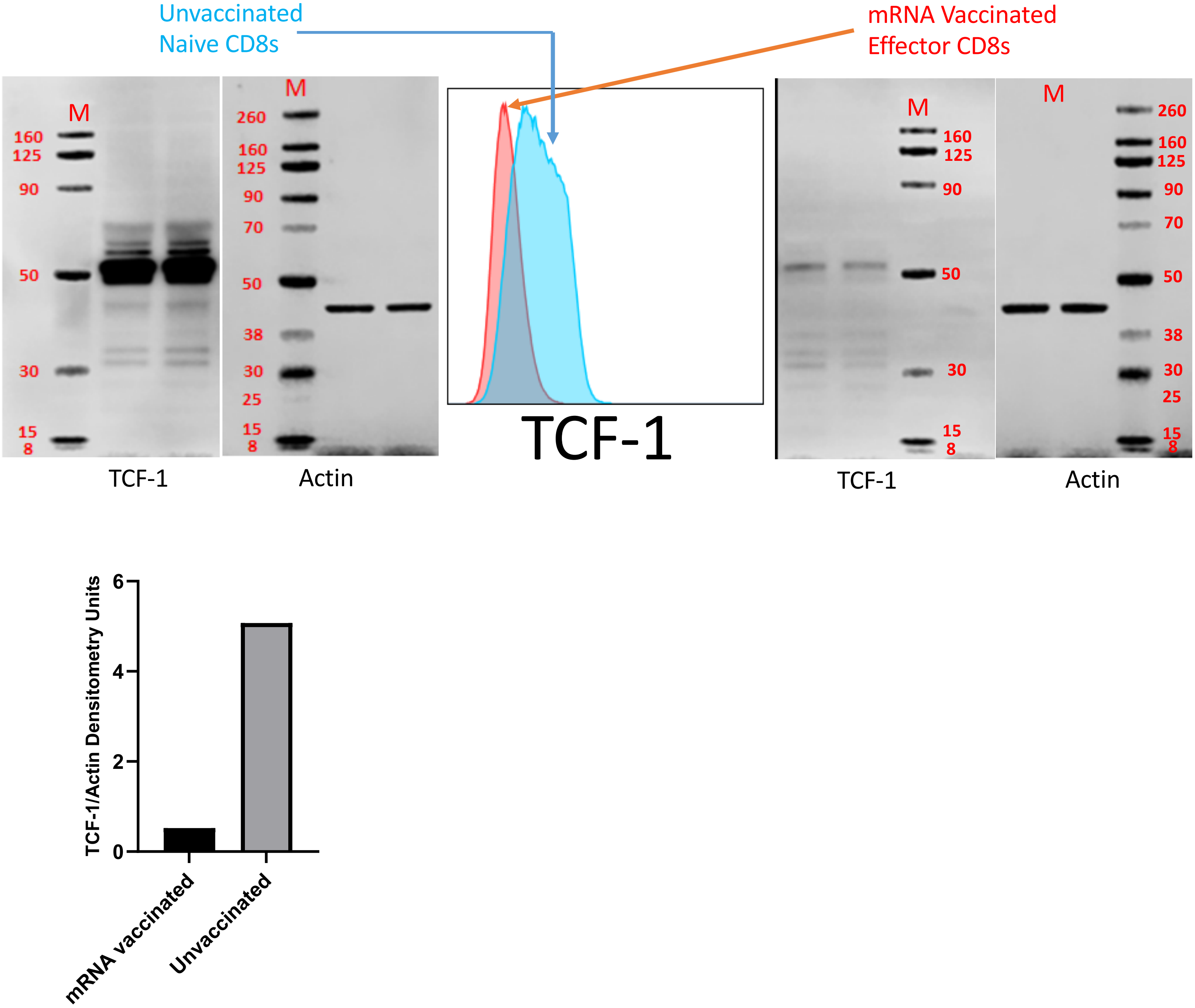
Unvaccinated
Naive CD8s
mRNA Vaccinated
Effector CD8s
M
160
125
90
50
30
15
8
260
70
38
25
TCF-1
Actin
TCF-1
TCF-1
Actin
TCF-1/Actin Densitometry Units
6
4
2
0
mRNA vaccinated
Unvaccinated

Sup. 3

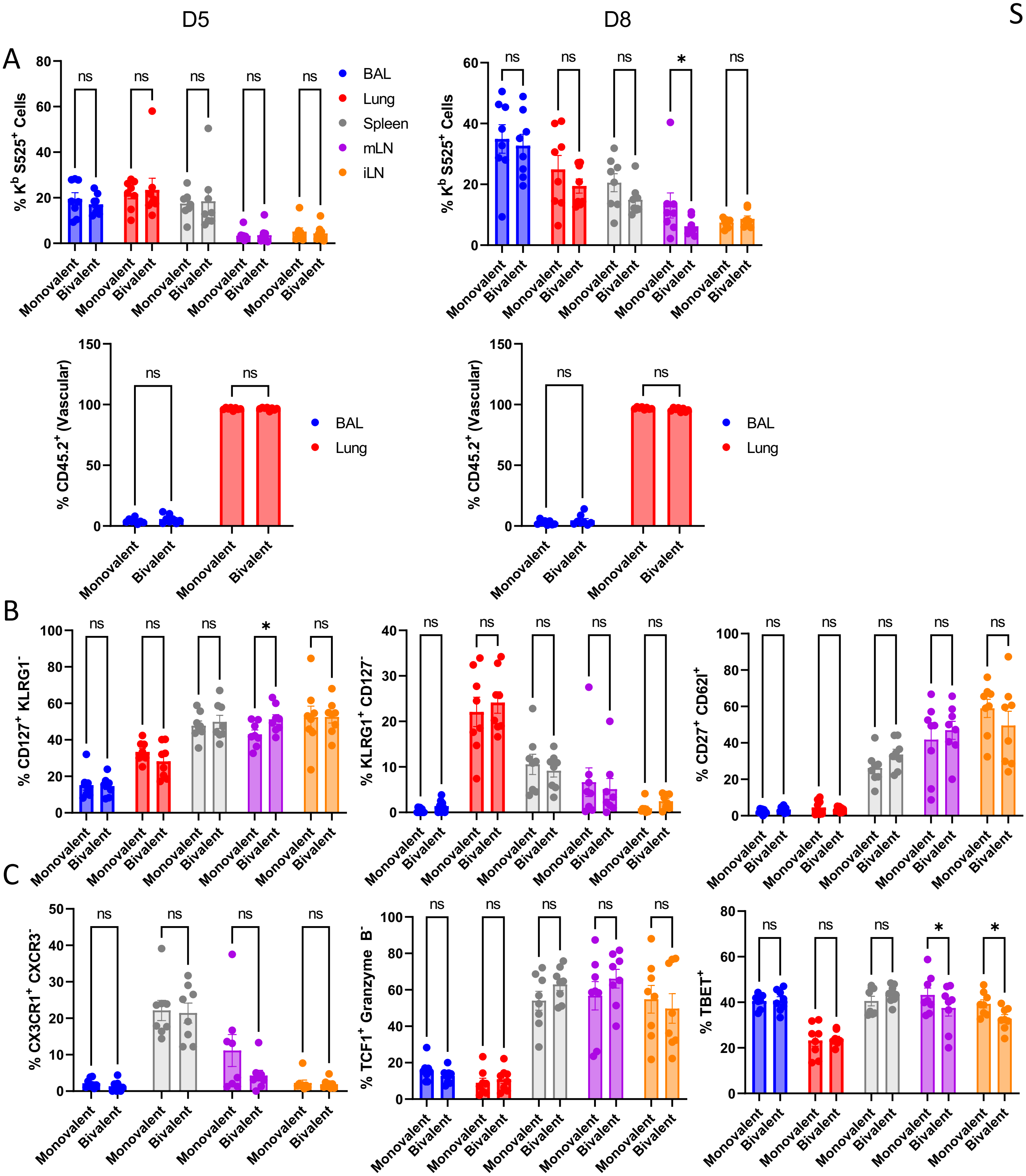
D5
D8
A
% K^b S525^+ Cells
BAL
Lung
Spleen
mLN
iLN
% CD45.2^+ (Vascular)
BAL
Lung
B
% CD127^+ KLRG1^-
% KLRG1^+ CD127^-
% CD27^+ CD62l^+
C
% CX3CR1^+ CXCR3^-
% TCF1^+ Granzyme B^-
% TBET^+
Monovalent
Bivalent
ns
*

Sup. 3

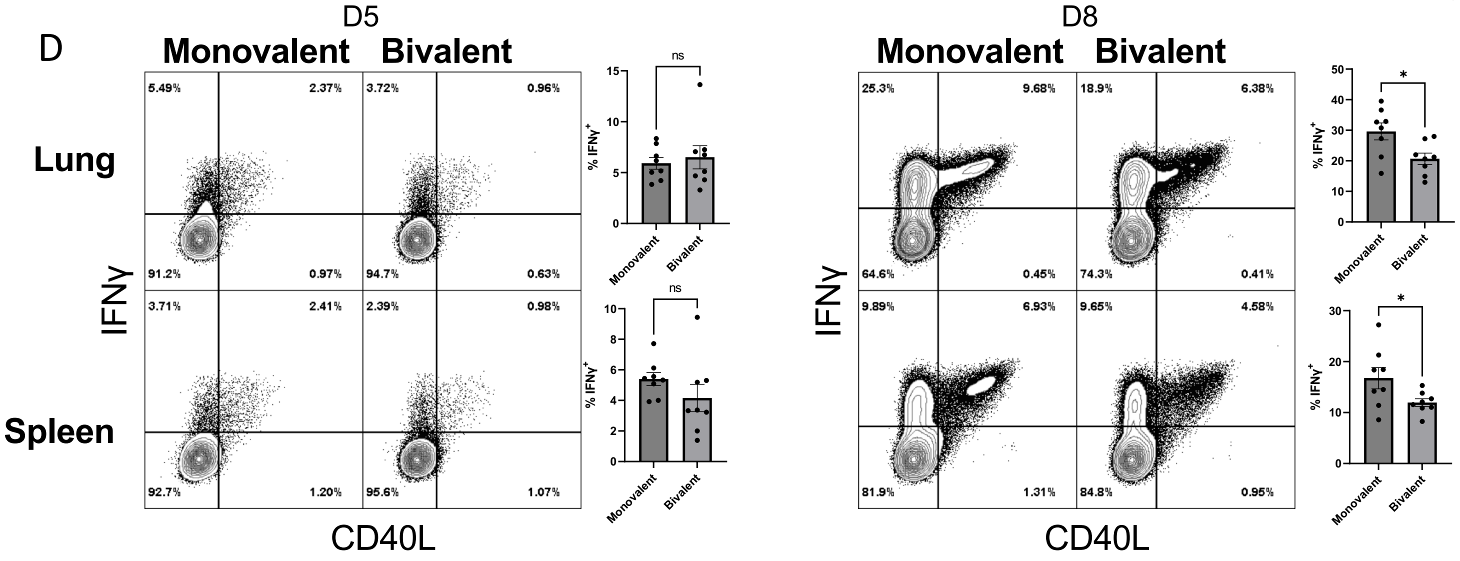
D
D5
Monovalent
Bivalent
Lung
Spleen
IFNγ
CD40L
5.49%
2.37%
91.2%
0.97%
3.72%
0.96%
94.7%
0.63%
3.71%
2.41%
92.7%
1.20%
2.39%
0.98%
95.6%
1.07%
ns
% IFNγ+
Monovalent
Bivalent
D8
25.3%
9.68%
64.6%
0.45%
18.9%
6.38%
74.3%
0.41%
9.89%
6.93%
81.9%
1.31%
9.65%
4.58%
84.8%
0.95%
*

Sup. 4

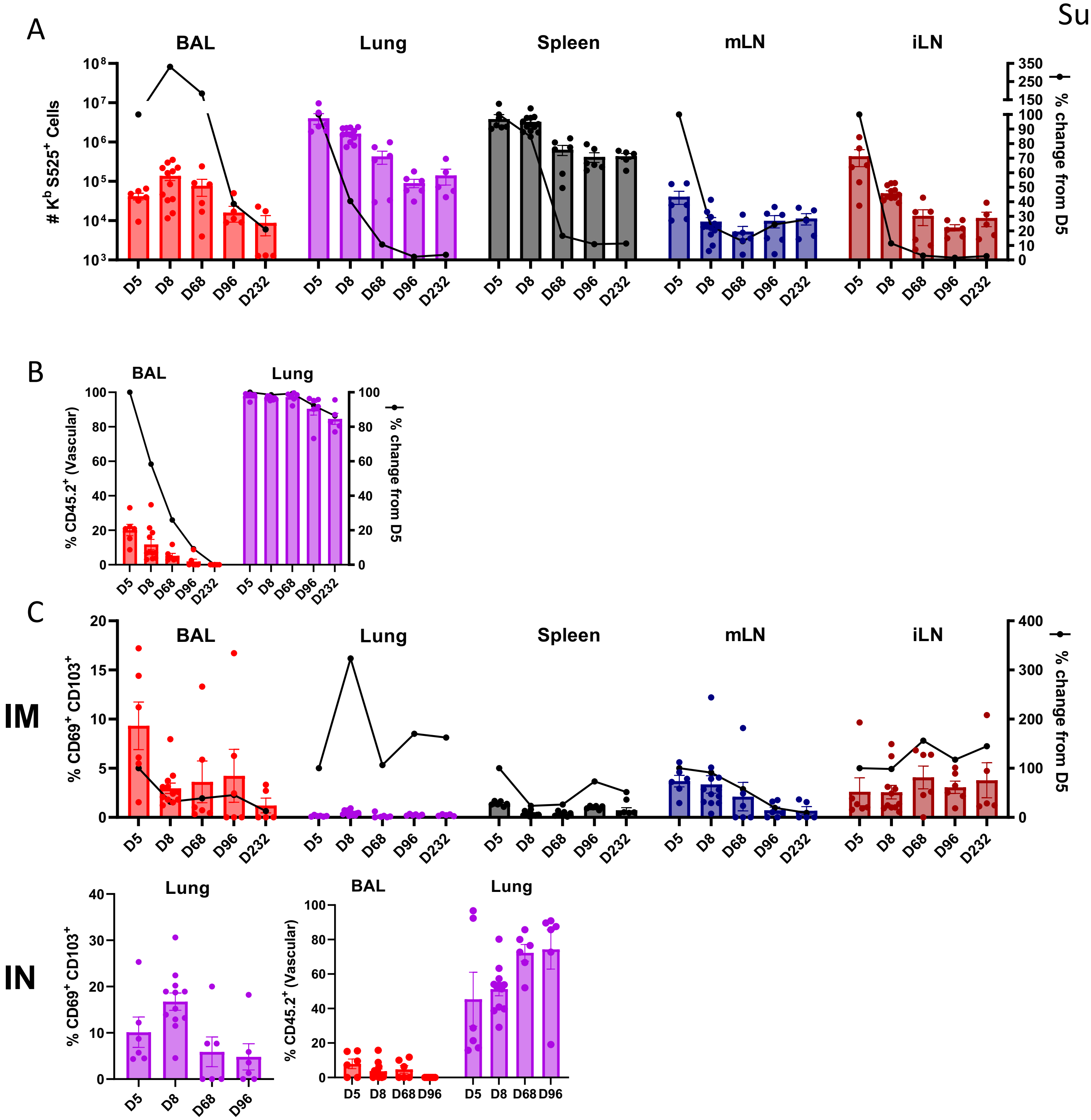

A
BAL
Lung
Spleen
mLN
iLN
K^b S525^+ Cells
% change from D5
D5 D8 D68 D96 D232
B
BAL
Lung
% CD45.2^+ (Vascular)
% change from D5
C
IM
% CD69^+ CD103^+
IN
% CD69^+ CD103^+
% CD45.2^+ (Vascular)
D5 D8 D68 D96

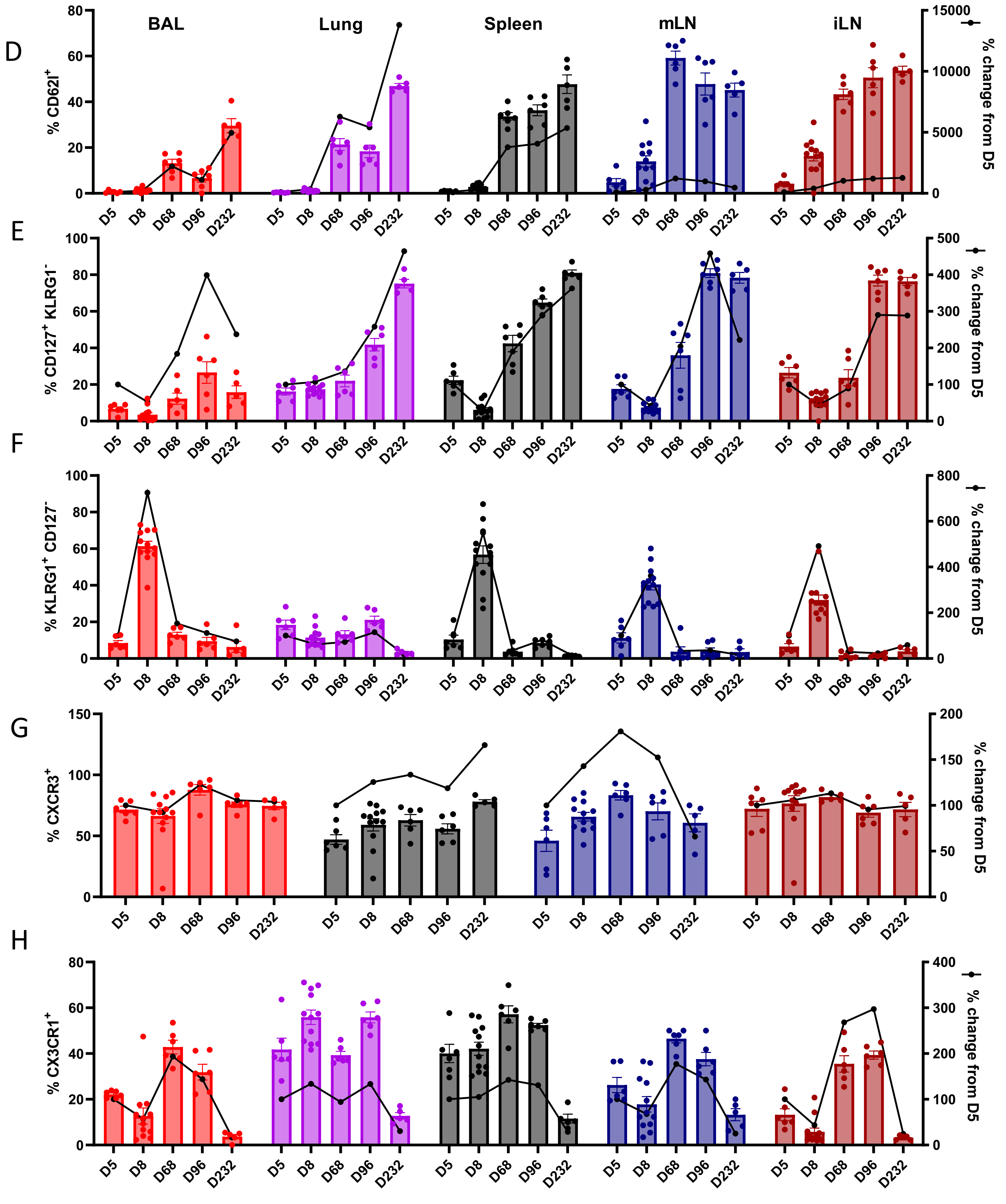
D
E
F
G
H
BAL
Lung
Spleen
mLN
iLN
% CD62l+
% CD127+ KLRG1-
% KLRG1+ CD127-
% CXCR3+
% CX3CR1+
% change from D5
D5
D8
D68
D96
D232

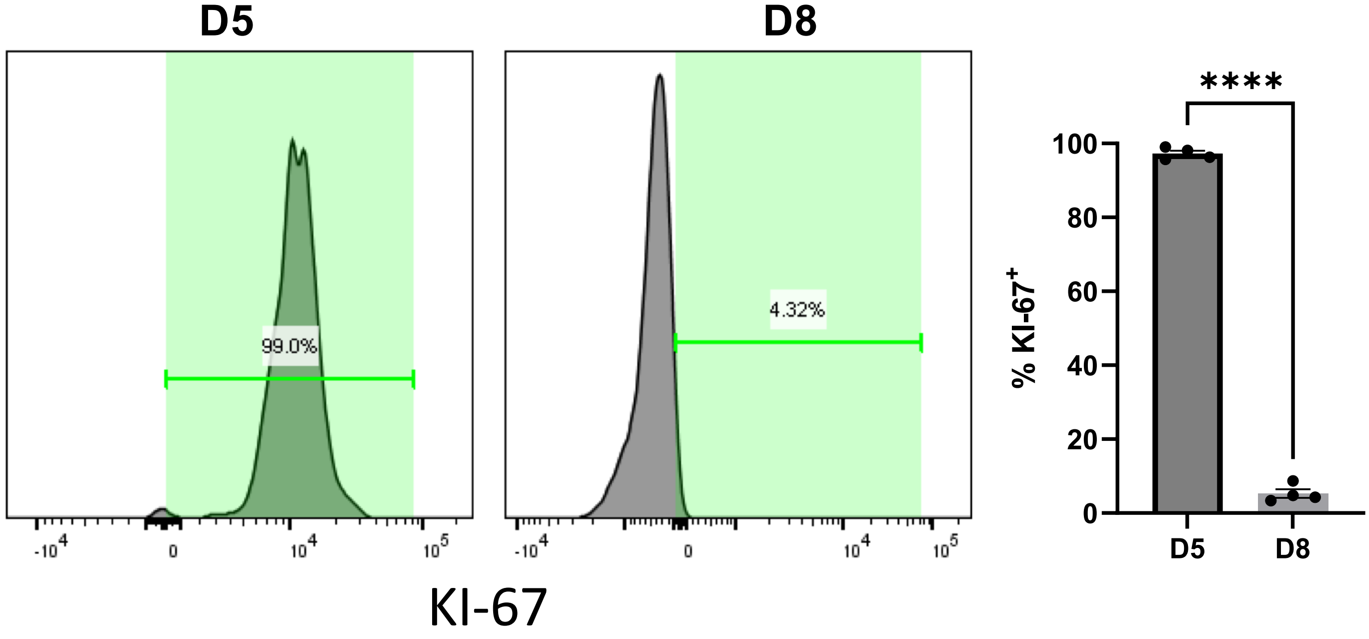
D5
D8
99.0%
4.32%
-10^4
0
10^4
10^5
KI-67
****
% KI-67+
100
80
60
40
20
0
D5
D8

Sup. 6

A

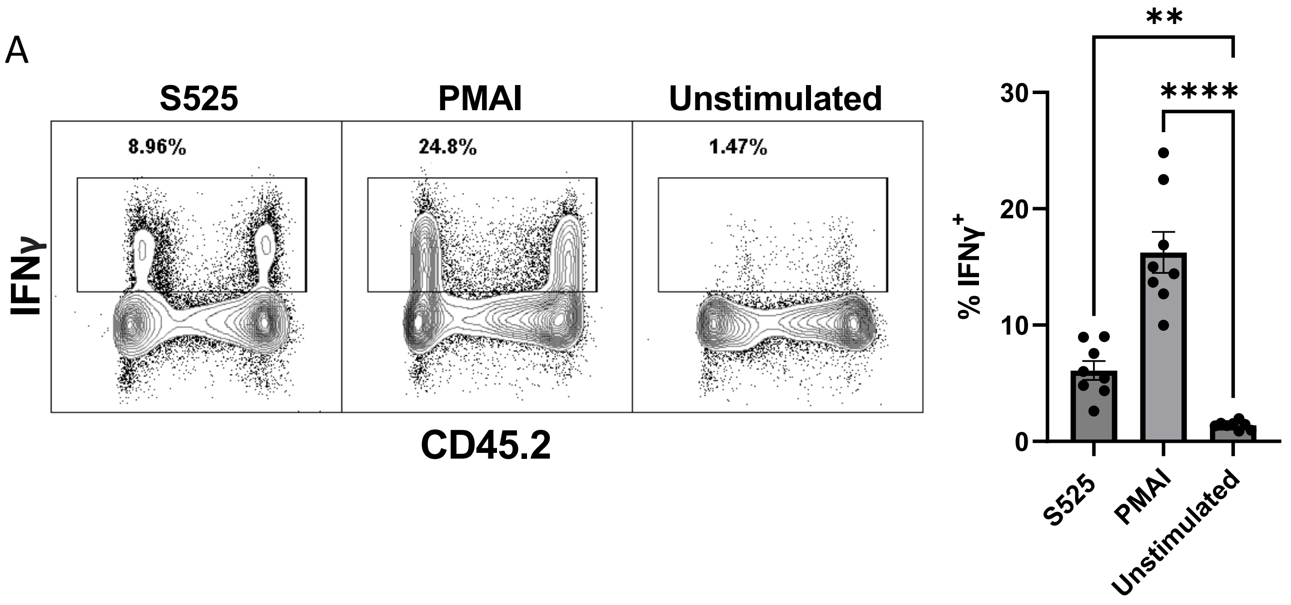
S525
PMAI
Unstimulated
8.96%
24.8%
1.47%
IFNγ
CD45.2
% IFNγ+
30
20
10
0
**
****
S525
PMAI
Unstimulated

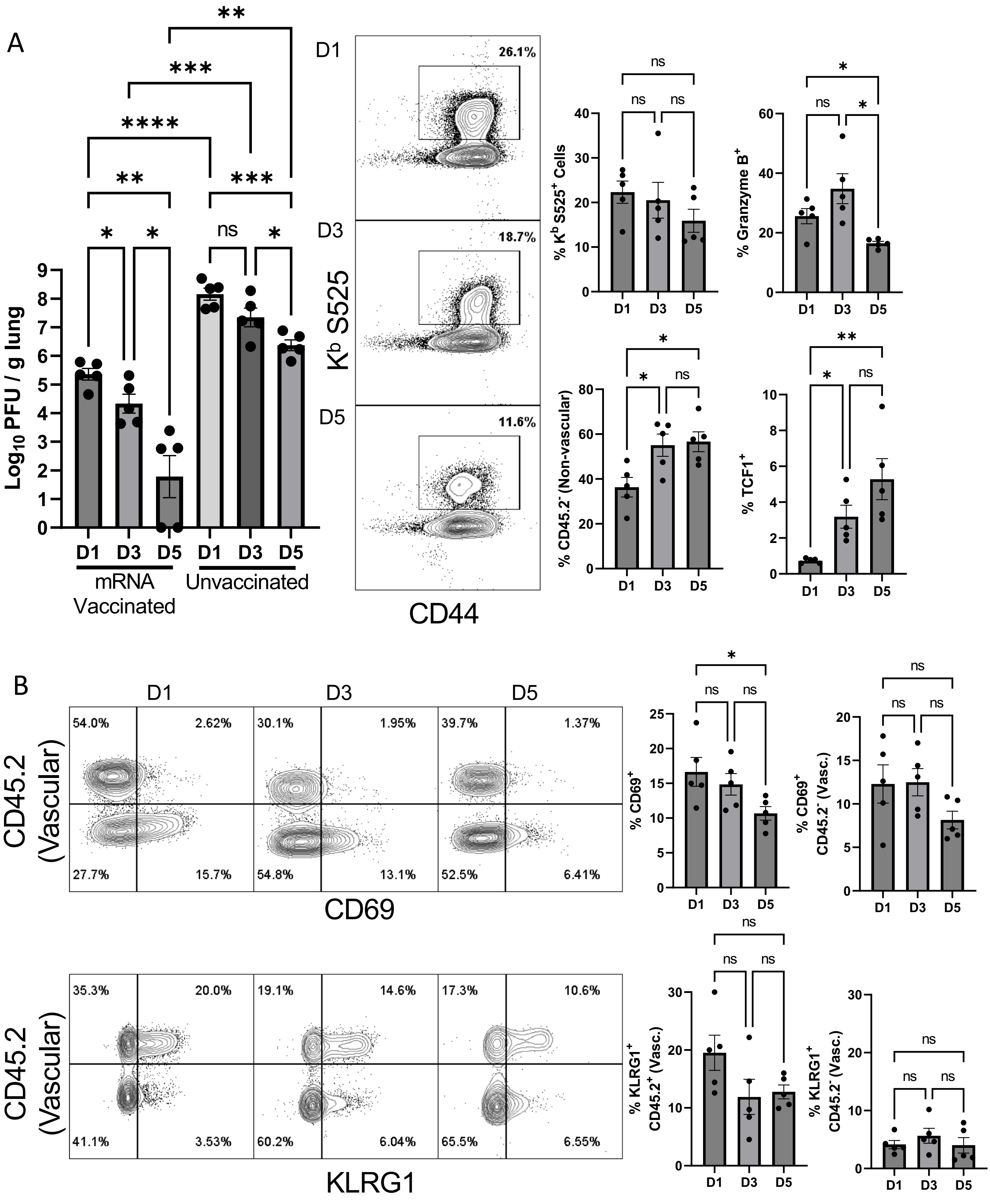
A
Log$_{10}$ PFU / g lung
D1 D3 D5 D1 D3 D5
mRNA Vaccinated
Unvaccinated
**
***
****
**
***
*
*
ns
*
D1
D3
D5
26.1%
18.7%
11.6%
K^b S525
CD44
% K^b S525$^+$ Cells
% Granzyme B$^+$
% CD45.2$^-$ (Non-vascular)
% TCF1$^+$
B
D1
D3
D5
54.0% 2.62% 27.7% 15.7%
30.1% 1.95% 54.8% 13.1%
39.7% 1.37% 52.5% 6.41%
CD45.2 (Vascular)
CD69
% CD69$^+$
% CD69$^+$ CD45.2$^-$ (Vasc.)
35.3% 20.0% 41.1% 3.53%
19.1% 14.6% 60.2% 6.04%
17.3% 10.6% 65.5% 6.55%
KLRG1
% KLRG1$^+$ CD45.2$^+$ (Vasc.)
% KLRG1$^+$ CD45.2$^-$ (Vasc.)

Sup. 8

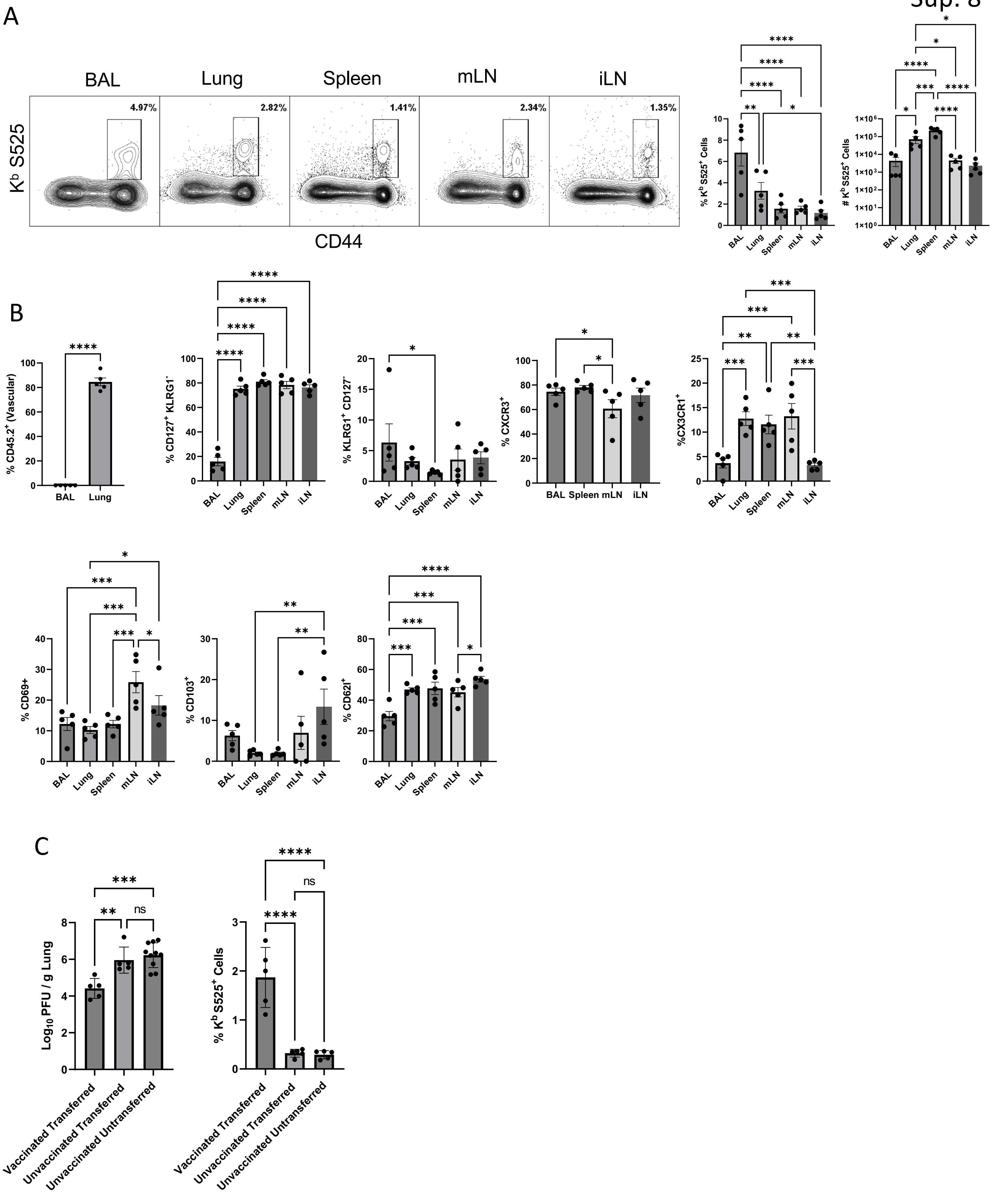
A
BAL
Lung
Spleen
mLN
iLN
4.97%
2.82%
1.41%
2.34%
1.35%
K^b S525
CD44
% K^b S525^+ Cells
# K^b S525^+ Cells
B
% CD45.2^+ (Vascular)
% CD127^+ KLRG1^-
% KLRG1^+ CD127^-
% CXCR3^+
%CX3CR1^+
% CD69+
% CD103^+
% CD62l^+
C
Log10 PFU / g Lung
% K^b S525^+ Cells
Vaccinated Transferred
Unvaccinated Transferred
Unvaccinated Untransferred
ns

Tissue processing and Flow cytometry

Spleens and lungs were processed into single cell suspensions using mechanical digestion and standard collagenase-based methods, as previously described (1). To stain for surface markers, single-cell suspensions were first stained for viability with Ghost Dye™ Red 780 (Tonbo Biosciences, stained with antibodies and tetramers diluted in Brilliant Stain Buffer (BSB, BD Biosciences) for 60 minutes at 4C, and fixed with 2% paraformaldehyde. All samples were acquired on LSRFortessa (BD Biosciences) and analyzed with FlowJo V.10 software (TreeStar, Ashland, OR).

Intracellular staining for transcription factors

To stain for intracellular factors directly *ex vivo*, single-cell suspensions were stained for viability as above. Next, samples were stained with antibodies and tetramers diluted in BSB (BD Biosciences), which were then fixed, permeabilized and subsequently stained for using the transcription factors staining kit (eBioscience) with the antibodies (indicated in **Supplemental Table 1)** diluted in eBioscience Perm Wash buffer. All samples were acquired on LSRFortessa (BD Biosciences) and analyzed with FlowJo V.10 software (TreeStar, Ashland, OR).

Intracellular Cytokine Staining (ICCS)

For intracellular cytokine staining, cells were plated and stimulated for 5 hours at 37C in the presence of brefeldin A (1 µl/ml, GolgiPlug, BD Biosciences), human recombinant IL-2 (10 U/well) and with or without S525 (Genscript) at 1ug/ml. After stimulation, cells were transferred to 96-well round bottom plate (Corning) and stained for viability dye as above, and fixed/permeabilizated with Cytofix/Cytoperm kit (BD Biosciences, Franklin Lakes, NJ) according to manufacturer's protocol. Samples were stained with antibodies corresponding to cytokines indicated in **Supplemental Table 1** in perm wash buffer. All the staining procedures were performed on ice.

Cells and Viruses

African Green Monkey Kidney Cells (Vero) were obtained from ATCC (ATCC; Manassas, VA, USA). The SARS-CoV-2 MA10/B.1.351 virus generated by and obtained from Dr. Ralph Baric (University of North Carolina-Chapel Hill) was tittered and propagated in Vero cells. SARS-CoV-2 viral titrations were performed by infecting Vero cell monolayers with serially diluted samples which were then incubated for 5 days, fixed, and plaque forming units were calculated from observing cytopathic effects under a light microscope.

Viral challenge

For all challenge studies, mice were euthanized on day 5 post challenge and lungs collected for viral titers (left lobe), histology, (a section of the inferior lobe) and T cell analysis (remainder of lung tissue). For virus titration, lungs were weighed, then homogenized in Opti-MEM media containing 3% FBS via bead beating, clarified by centrifugation and titrated as described above. Lung sections for histology were taken from uninflated lungs and fixed in 10% neutral buffered formalin, sectioned, and stained with Hematoxylin and Eosin (H&E) by conventional methods.

1. C. B. Marinaik *et al.*, Programming Multifaceted Pulmonary T Cell Immunity by Combination Adjuvants. *Cell Rep Med* **1**, 100095 (2020).
2. M. A. Croyle, D. J. Anderson, B. J. Roessler, G. L. Amidon, Development of a highly efficient purification process for recombinant adenoviral vectors for oral gene delivery. *Pharmaceutical development and technology* **3**, 365-372 (1998).
3. Y. Ambuel *et al.*, A rapid immunization strategy with a live-attenuated tetravalent dengue vaccine elicits protective neutralizing antibody responses in non-human primates. *Front Immunol* **5**, 263 (2014).

Supplementary Table 1. List of antibodies used in the manuscript

Antibodies used at 1:200 concentration unless otherwise indicated

Antibody (Dilution Factor)	Company	Catalogue number
Rat anti-mouse CD8α-BUV395-	BD Biosciences	563786
Rat anti-mouse CD4-BUV496-	BD Biosciences	564667
Rat anti-mouse CD44-BV510-	BD Biosciences	563114
Rat anti-mouse CD62L-PE-CF594-	BD Biosciences	562404
Hamster anti-mouse-CD69-PE-Cy7-	BD Biosciences	553237
Hamster Anti-KLRG1-BV711-	BD Biosciences	564014
Rat anti-mouse IFN-γ-APC- (1:400)	BD Biosciences	554413
Rat anti-CD127-BV605-	Biolegend	135041
Mouse anti-CX3CR1-BV785-	Biolegend	SA011F11
Hamster Anti-CD49a-BV605-	BD Pharmingen	740375
Hamster Anti-KLRG1-BV711-	BD Pharmingen	564014
Rat Anti-CD62L-Alexa 700-	BD Pharmingen	560517
Hamster Anti-CD279 (PD-1)-BV 650-	BD Pharmingen	744546
Hamster Anti-Mouse CD69-PE-Cy7	BD Pharmingen	552879
Rat Anti- CD4-APC-	BD Pharmingen	553051
Hamster Anti- CD103 Antibody-FITC	eBioscience	11-1031-85
Mouse anti-CD45.2 Monoclonal Antibody	eBioscience	12-0454-82
Mouse anti-Granzyme B-PE 1:10	Thermo Fisher Scientific	MHGB04
Rat anti-CD127-BV650- 1:150	Biolegend	135043
Mouse anti-CX3CR1-BV785	Biolegend	SA011F11

APC H2-K^b tetramers bearing the Spike peptide S525 1:150	NIH Tetramer Core Facility at Emory University	N/A
BV421 HLA-A*02:01 Tetramers bearing the Spike peptides: 269-277 YLQPRTFLL, or 1000-1008 RLQSLQTYV	NIH Tetramer Core Facility at Emory University	
